# Two new cases of polymorphism in diagonally substituted rubrene derivatives

**DOI:** 10.1107/S2056989023002736

**Published:** 2023-03-31

**Authors:** Margaret L. Clapham, Christopher J. Douglas

**Affiliations:** a 207 Pleasant St SE, Minneapolis, MN, 55455, USA; Texas A & M University, USA

**Keywords:** rubrene, polymorphism, crystal structure

## Abstract

The crystal structures of two rubrene derivatives, 5,11-diphenyl-6,12-bis­[4-(tri­fluoro­meth­yl)phen­yl]tetra­cene, C_44_H_26_F_6_, and 5,11-bis­(4-*tert*-butyl­phen­yl)-6,12-di­phenyl­tetra­cene, C_50_H_44_, are presented. Each are substituted on diagonal (5/11) phenyl rings. Each derivative has one polymorph reported previously. A discussion of the differences between each derivative and its previously reported polymorph is provided. The triclinic packing of the CF_3_-substituted structure is similar to the packing of the parent rubrene’s triclinic polymorph. In the *tert*-butyl-substituted structure, a planar tetra­cene core formed, which has been hypothesized but never published. Crystallization conditions are provided as they differ from previous reports.

## Abstract 2

Two new cases of polymorphism in two different substituted rubrenes are reported. These are some of the first examples in rubrene derivatives of polymorphism occurring in a separate crystal class.

## 1. Chemical context

Rubrene (5,6,11,12-tetra­phenyl­tetra­cene) has been widely studied in the literature for its excellent electronic properties. Many synthetic attempts have been made to alter the mol­ecular structure in the hope of improving these properties (Uttiya *et al.*, 2014 ▸; Ogden *et al.*, 2017 ▸; Paraskar *et al.*, 2008 ▸). Mol­ecular tuning of these derivatives has led to unpredictable crystal packing. While some derivatives have been reported to form the ideal herringbone crystal structure, others have not, with no reported structures exhibiting polymorphism in different crystal classes similar to the parent rubrene. The rubrene library has grown significantly over the years and now includes over 35 derivatives in a variety of crystalline arrangements (Clapham *et al.*, 2021 ▸). This library has provided an enticing database for computational scientists looking to add predictability and reasoning to crystal-packing formation (Sutton *et al.*, 2015 ▸). We wish to add to this rubrene library two additional structures. They are not polymorphs of each other, but instead polymorphs of previously published compounds. 
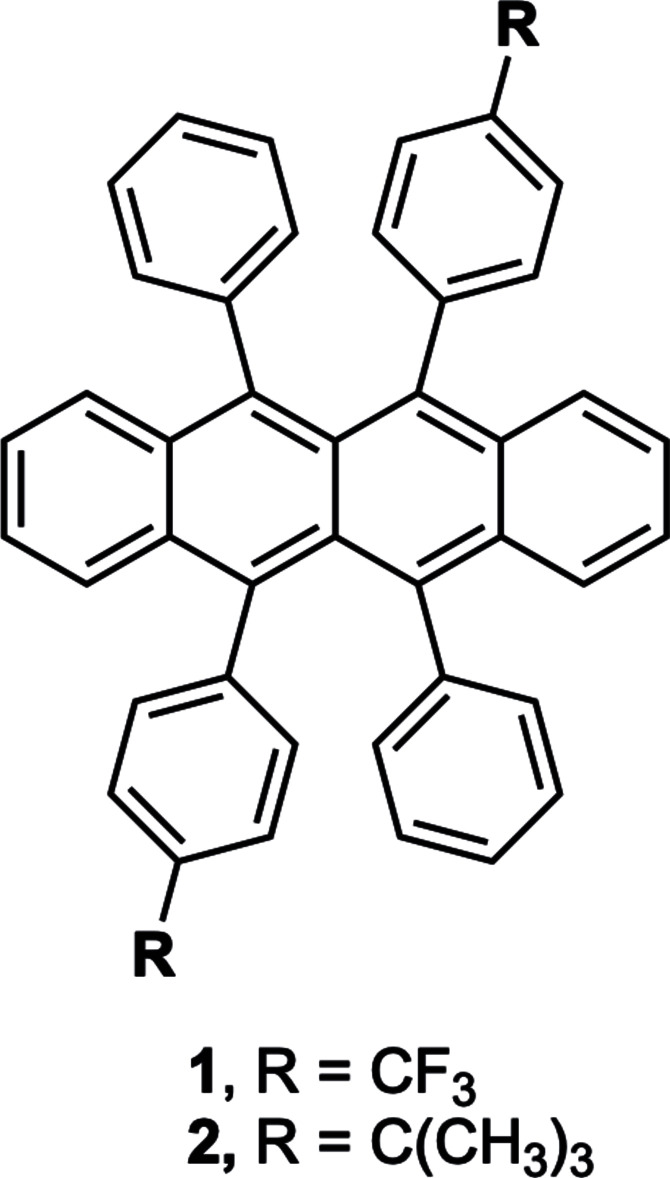


We report here new crystal packing for each compound, making these some of the first cases of polymorphism in rubrene derivatives. This report serves two purposes: the first as a caution to synthetic chemists that polymorphism can and does exist in these materials, even if it has not been published, as is the case for 5,11-bis­(4-tri­fluoro­methyl­phen­yl)-6,12-bi­phenyl­tetra­cene (compound **1**). The second purpose serves as an encouragement to explore more of the rubrene library in future studies. For example, 5,11-bis­(4-*tert*-butyl­phen­yl)-6,12-bi­phenyl­tetra­cene (compound **2**) has been largely overlooked, despite its promising carrier mobility (Haas *et al.*, 2007 ▸), likely because no crystal structure with the ideal herringbone formation had been reported in the database.

## 2. Structural commentary

**Figure 1:**
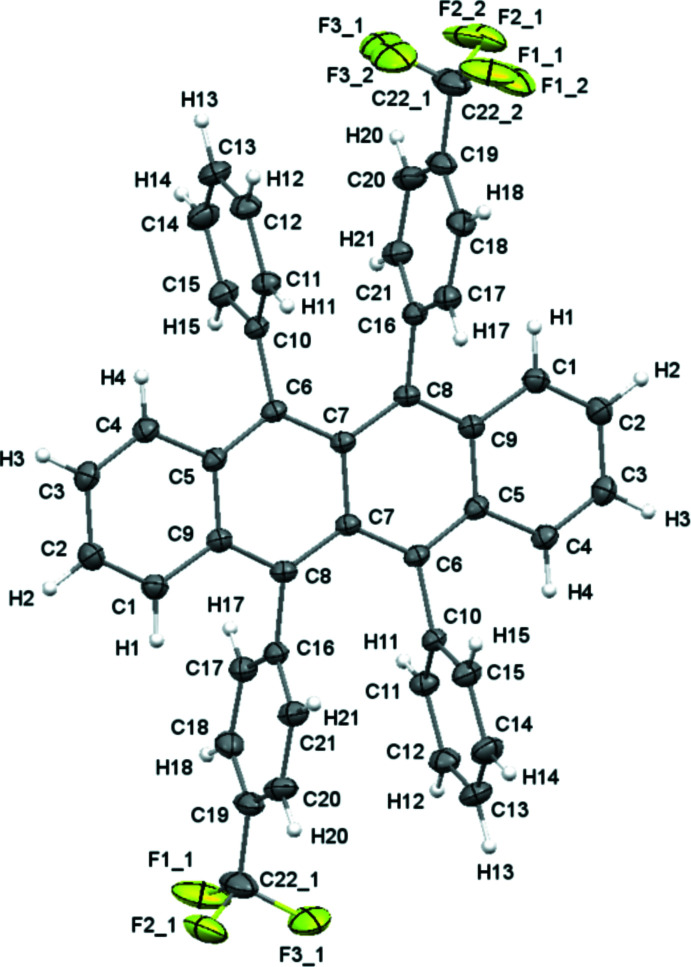
Crystal structure of **1** with displacement ellipsoids shown at the 50% probability level.

Both rubrene mol­ecules in this report have been reported and synthesized previously (Haas *et al.*, 2007 ▸; Uttiya *et al.*, 2014 ▸). Each contains substitutions on the 5 and 11 peripheral phenyl rings. Many rubrene derivatives are shown to twist along the tetra­cene core in the solid state, such as the first polymorph of **2**. Here, both derivatives display planar tetra­cene backbones (Figs. 1 ▸ and 2 ▸).

## 3. Supra­molecular features

**Figure 3:**
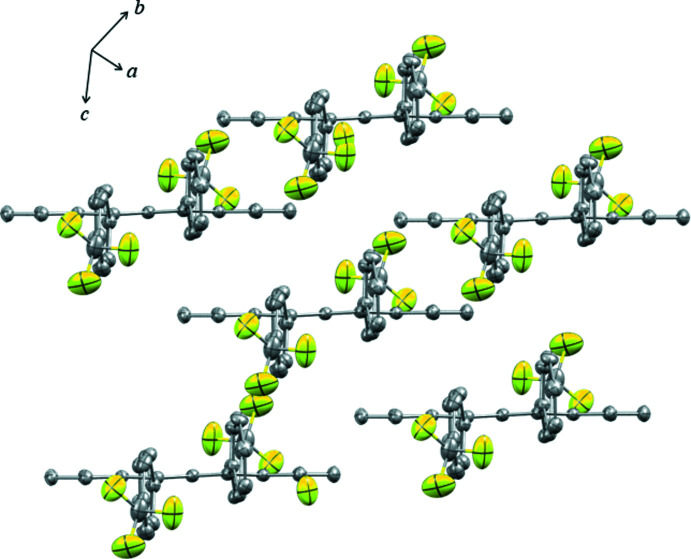
Crystal projection of **1** displaying brick-like packing.

**Figure 4:**
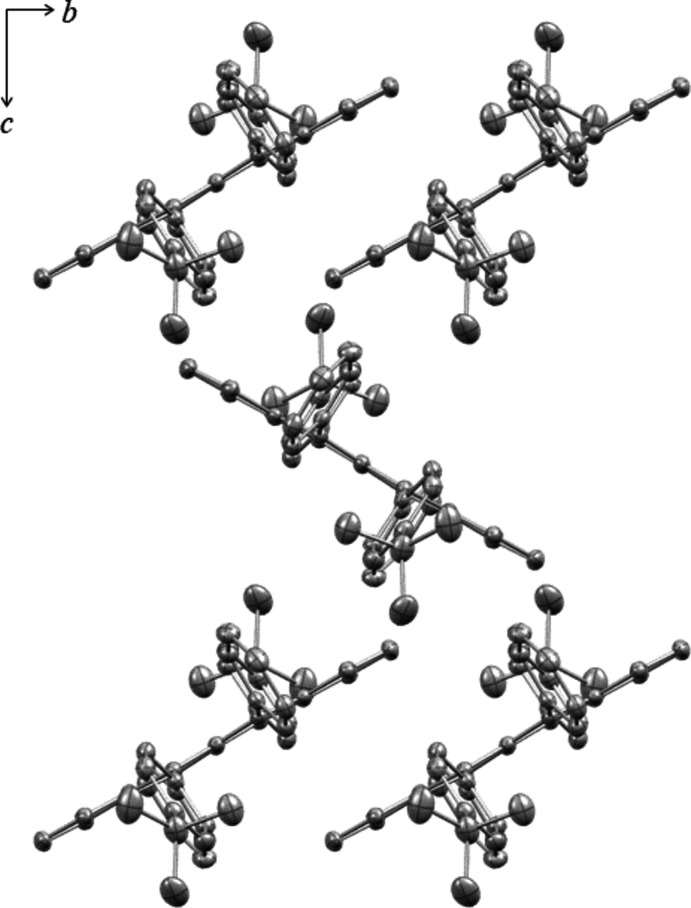
Crystal projection of **2** displaying herringbone packing.

Compound **1** packs in a brick-like arrangement (Fig. 3 ▸), similar in structure to triclinic rubrene. This arrangement displays π-stacking inter­actions of the tetra­cene cores. This is contrasted with the herringbone arrangement of **2** (Fig. 4 ▸). While there exist sets of π-stacking dimers, alternating layers are rotated, creating the ‘z’ or herringbone arrangement.

## 4. Database survey

Previously, only the monoclinic compound **1** had been reported (CSD CIYXUF; Uttiya *et al.*, 2014 ▸). The structure displays a planar tetra­cene core with the desired herringbone packing, similar to rubrene. Additionally, like rubrene, we now report a triclinic form. While the triclinic form retains the planar backbone, it packs in a brick-like arrangement, which has been shown with rubrene to have significantly reduced charge mobility (Matsukawa *et al.*, 2010 ▸). This is a similar case to the NO_2_-substituted rubrene derivative [5,11-bis­(4-nitro­phen­yl)-6,12-bi­phenyl­tetra­cene] in which the monoclinic form was discovered (Uttiya *et al.*, 2014 ▸), with the triclinic reported later (Moret & Gavezzotti, 2022 ▸).

This instance of polymorphs with differing carrier mobility was also seen for the previously published structure of **2** (Schuck *et al.*, 2007 ▸). Schuck *et al.* reported two crystalline forms; however, full structural analysis was only able to be carried out on the monoclinic form (CSD PIFHOC). While it was noted that the published monoclinic structure had no carrier mobility, the second morphology had a high measured mobility. As a result of the mobility and *d*-spacing measurements, it was hypothesized this mol­ecule took on a herringbone arrangement. We have therefore now performed a full structural characterization, and confirmed the herringbone arrangement of **2** as hypothesized.

## 5. Synthesis and crystallization

The synthesis of **1** was published previously (Uttiya *et al.*, 2014 ▸). The authors reported crystal growth in acetone; however, attempts at crystallization with acetone either by cooling or through evaporation were unsuccessful in growing the monoclinic structure previously reported. Any crystals obtained through this method, other solvent mixtures (ethanol, methanol, DCM:methanol), or physical vapor transport (PVT) all produced the triclinic form reported here, thus necessitating this publication to serve as a cautionary notice.

Synthetic and crystallization procedures of **1** were followed for **2**. In contrast to the PVT methods previously reported (Haas *et al.*, 2007 ▸), we found both polymorphs to be grown by solution methods, with the herringbone polymorph in the minority. Compound **2** was dissolved in a minimal amount of DCM and layered with methanol, in an approximate 1:3 ratio. We observed two different morphologies: the monoclinic structure in thin sheets as previously reported, as well as some dark-red thin plates. Likely due to improved instrumentation in more recent years, we were able to collect full structural data on the thin plates. We also note that the herringbone polymorph has excellent air stability. Whereas the monoclinic polymorph oxidizes when exposed to air, the herringbone polymorph remains stable for many months and retains its dark-red color, making it easily distinguishable from the other polymorph.

## 6. Refinement

Crystal data, collection and structure refinement details are summarized in Table 1 ▸ for compound **1** (C_44_H_26_F_6_) along with the previously published polymorph (CIYXUF) and compound **2** (C_50_H_44_) with the previously published polymorph (PIFHOC) for comparison.

## Supplementary Material

Crystal structure: contains datablock(s) 1, 2, CIYXUF, PIFHOC, global. DOI: 10.1107/S2056989023002736/jy2028sup1.cif

Supporting information file. DOI: 10.1107/S2056989023002736/jy20281sup4.cml

Structure factors: contains datablock(s) 1. DOI: 10.1107/S2056989023002736/jy20281sup7.hkl

Supporting information file. DOI: 10.1107/S2056989023002736/jy20282sup5.cml

Structure factors: contains datablock(s) 2. DOI: 10.1107/S2056989023002736/jy20282sup8.hkl

Supporting information file. DOI: 10.1107/S2056989023002736/jy2028PIFHOCsup6.cml

CCDC references: 651431, 970522

Additional supporting information:  crystallographic information; 3D view; checkCIF report

## Figures and Tables

**Figure 2:**
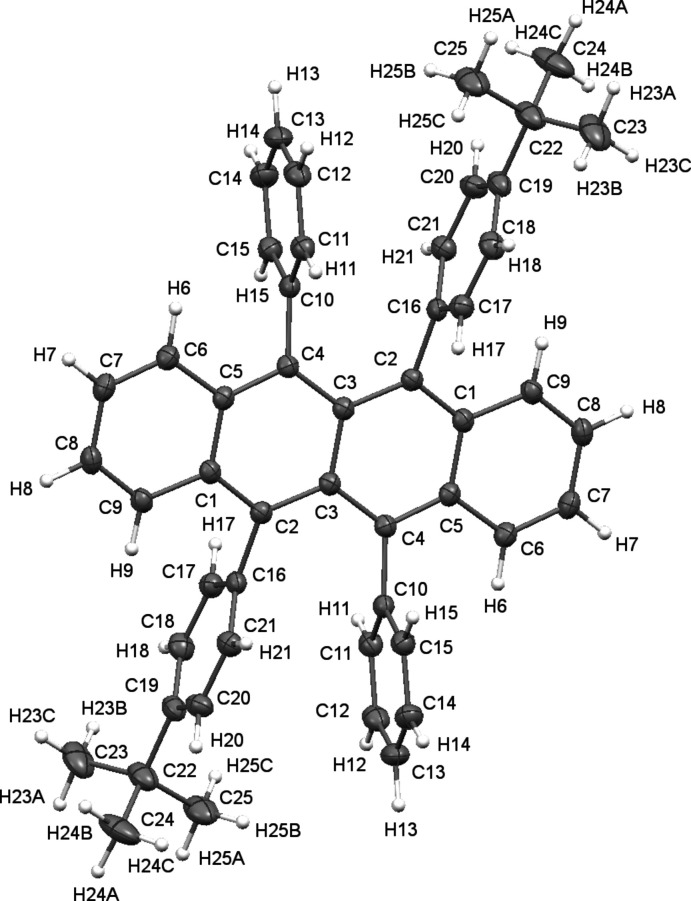
Crystal structure of **2** with displacement ellipsoids shown at the 50% probability level.

**Table 1:** Experimental details

	**1**	CIYXUF	**2**	PIFHOC
Crystal data				
Chemical formula	C_44_H_26_F_6_	C_44_H_26_F_6_	C_50_H_44_	C_50_H_44_
*M*_r_	668.68	668.68	644.85	644.85
Crystal system, space group	Triclinic, *P* 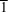	Monoclinic, *P*2_1_/*c*	Monoclinic, *P*2_1_/*c*	Monoclinic, *P*2_1_/*c*
Temperature (K)	125	123	100	292
*a*, *b*, *c* (Å)	7.0808 (3), 8.3978 (4), 13.4212 (5)	15.9782 (6), 7.2762 (2), 13.9814 (6)	17.4565 (15), 7.2014 (6), 13.9356 (12)	23.527 (3), 9.0277 (10), 17.764 (2)
α, β, γ (°)	88.234 (2), 80.559 (1), 81.623 (2)	90, 102.701 (2), 90	90, 92.593 (2), 90	90, 95.928 (4), 90
*V* (Å^3^)	778.84 (6)	1585.71 (10)	1750.1 (3)	3752.8 (8)
*Z*	1	2	2	4
Radiation type	Mo *K*α	Mo *K*α	Cu *K*α	Mo *K*α
μ (mm^−1^)	0.11	0.11	0.52	0.06
Crystal size (mm)	0.25 × 0.08 × 0.08	0.35 × 0.28 × 0.10	0.50 × 0.25 × 0.10	0.36 × 0.16 × 0.04

Data collection				
Diffractometer	Bruker Photon-II CMOS	Rigaku RAXIS II	Bruker Photon-II CMOS	Brucker *SMART* CCD
Absorption correction	Multi-scan (*SADABS*; Krause *et al.*, 2015 ▸)	Numerical (*CrystalClear-SM Expert*; Rigaku, 2009 ▸)’	Multi-scan (*SADABS*; Krause *et al.*, 2015 ▸)	Multi-scan (*SADABS*; Krause *et al.*, 2015 ▸)
*T*_min_, *T*_max_	0.664, 0.746	0.971, 0.992	0.620, 0.754	0.990, 0.997
No. of measured, independent and observed [*I* > 2σ(*I*)] reflections	17779, 4796, 3777	15137, 3643, 3222	34891, 3549, 3431	31129, 6626, 3478
*R*_int_	0.033	0.051	0.034	0.100
(sin θ/λ)_max_ (Å^−1^)	0.719	0.650	0.626	0.596

Refinement				
*R*[*F* ^2^ > 2σ(*F* ^2^)], *wR*(*F* ^2^), *S*	0.046, 0.133, 1.03	0.066, 0.135, 1.11	0.041, 0.105, 1.08	0.098, 0.169, 1.11
No. of reflections	4796	3643	3549	6626
No. of parameters	254	197	229	536
No. of restraints	54	183	0	0
H-atom treatment	H-atom parameters constrained	H-atom parameters constrained	H-atom parameters constrained	H atoms treated by a mixture of independent and constrained refinement
Δρ_max_, Δρ_min_ (e Å^−3^)	0.36, −0.22	0.37, −0.30	0.23, −0.21	0.29, −0.21

Computer programs: *SMART* and *SAINT* (Bruker, 2000 ▸), *APEX3* and *SAINT* (Bruker, 2016 ▸), *CrystalClear-SM Expert* (Rigaku, 2009 ▸), *SHELXT2014/5* (Sheldrick, 2015*a* ▸), *SHELXS2013*, *SHELXS97*, *SHELXL97* and*SHELXTL* (Sheldrick, 2008 ▸), *SHELXL2013* and *SHELXL2018/3* (Sheldrick, 2015*b* ▸), *ShelXle* (Hübschle *et al.*, 2011 ▸), and *ORTEP-3 for Windows* and *WinGX* (Farrugia, 2012 ▸).

## supplementary crystallographic information

### 5,11-Diphenyl-6,12-bis[4-(trifluoromethyl)phenyl]tetracene (1) Crystal data

**Table:**

C_44_H_26_F_6_	*Z* = 1
*M**_r_* = 668.68	*F*(000) = 344
Triclinic, *P*1	*D*_x_ = 1.426 Mg m^−^^3^
*a* = 7.0808 (3) Å	Mo *K*α radiation, λ = 0.71073 Å
*b* = 8.3978 (4) Å	Cell parameters from 6944 reflections
*c* = 13.4212 (5) Å	θ = 2.5–30.6°
α = 88.234 (2)°	µ = 0.11 mm^−^^1^
β = 80.559 (1)°	*T* = 125 K
γ = 81.623 (2)°	Needle, red
*V* = 778.84 (6) Å^3^	0.25 × 0.08 × 0.08 mm

### 5,11-Diphenyl-6,12-bis[4-(trifluoromethyl)phenyl]tetracene (1) Data collection

**Table:**

Bruker Photon-II CMOS diffractometer	3777 reflections with *I* > 2σ(*I*)
Radiation source: micro	*R*_int_ = 0.033
φ and ω scans	θ_max_ = 30.7°, θ_min_ = 2.5°
Absorption correction: multi-scan (SADABS; Krause *et al.*, 2015)	*h* = −10→10
*T*_min_ = 0.664, *T*_max_ = 0.746	*k* = −12→12
17779 measured reflections	*l* = −19→19
4796 independent reflections	

### 5,11-Diphenyl-6,12-bis[4-(trifluoromethyl)phenyl]tetracene (1) Refinement

**Table:**

Refinement on *F*^2^	Primary atom site location: structure-invariant direct methods
Least-squares matrix: full	Secondary atom site location: difference Fourier map
*R*[*F*^2^ > 2σ(*F*^2^)] = 0.046	Hydrogen site location: inferred from neighbouring sites
*wR*(*F*^2^) = 0.133	H-atom parameters constrained
*S* = 1.03	*w* = 1/[σ^2^(*F*_o_^2^) + (0.0677*P*)^2^ + 0.203*P*] where *P* = (*F*_o_^2^ + 2*F*_c_^2^)/3
4796 reflections	(Δ/σ)_max_ < 0.001
254 parameters	Δρ_max_ = 0.36 e Å^−^^3^
54 restraints	Δρ_min_ = −0.22 e Å^−^^3^

### 5,11-Diphenyl-6,12-bis[4-(trifluoromethyl)phenyl]tetracene (1) Special details

**Table:**

Geometry. All esds (except the esd in the dihedral angle between two l.s. planes) are estimated using the full covariance matrix. The cell esds are taken into account individually in the estimation of esds in distances, angles and torsion angles; correlations between esds in cell parameters are only used when they are defined by crystal symmetry. An approximate (isotropic) treatment of cell esds is used for estimating esds involving l.s. planes.

### 5,11-Diphenyl-6,12-bis[4-(trifluoromethyl)phenyl]tetracene (1) Fractional atomic coordinates and isotropic or equivalent isotropic displacement parameters (Å^2^)

**Table:**

	*x*	*y*	*z*	*U*_iso_*/*U*_eq_	Occ. (<1)
C1	0.01792 (16)	0.39026 (13)	0.66732 (8)	0.0226 (2)	
H1	−0.009095	0.444837	0.730124	0.027*	
C2	−0.10396 (16)	0.28909 (14)	0.64654 (9)	0.0258 (2)	
H2	−0.215225	0.274960	0.694302	0.031*	
C3	−0.06545 (17)	0.20452 (14)	0.55371 (9)	0.0256 (2)	
H3	−0.151890	0.135159	0.539123	0.031*	
C4	0.09514 (16)	0.22274 (13)	0.48564 (8)	0.0226 (2)	
H4	0.120443	0.163455	0.424577	0.027*	
C5	0.22728 (15)	0.32896 (12)	0.50350 (8)	0.0190 (2)	
C6	0.39655 (15)	0.34315 (12)	0.43500 (7)	0.0182 (2)	
C7	0.52091 (14)	0.45501 (12)	0.45239 (7)	0.0175 (2)	
C8	0.68520 (15)	0.48264 (12)	0.38051 (7)	0.0184 (2)	
C9	0.81357 (15)	0.58334 (12)	0.40331 (8)	0.0188 (2)	
C10	0.44475 (15)	0.22325 (12)	0.35087 (8)	0.0196 (2)	
C11	0.36977 (16)	0.24829 (13)	0.26079 (8)	0.0220 (2)	
H11	0.290420	0.346631	0.250057	0.026*	
C12	0.41022 (17)	0.13050 (14)	0.18663 (9)	0.0261 (2)	
H12	0.359656	0.149270	0.125314	0.031*	
C13	0.52406 (19)	−0.01412 (14)	0.20194 (9)	0.0299 (3)	
H13	0.552752	−0.093917	0.150907	0.036*	
C14	0.5959 (2)	−0.04202 (14)	0.29183 (10)	0.0307 (3)	
H14	0.672738	−0.141528	0.302808	0.037*	
C15	0.55537 (17)	0.07591 (13)	0.36619 (9)	0.0252 (2)	
H15	0.603751	0.055584	0.428018	0.030*	
C16	0.72284 (15)	0.42311 (13)	0.27387 (8)	0.0201 (2)	
C17	0.64437 (17)	0.51947 (14)	0.20039 (8)	0.0239 (2)	
H17	0.560887	0.616515	0.219579	0.029*	
C18	0.68658 (18)	0.47556 (15)	0.09905 (9)	0.0281 (2)	
H18	0.632752	0.542318	0.049352	0.034*	
C19	0.80767 (17)	0.33375 (16)	0.07142 (9)	0.0288 (3)	
C20	0.88955 (17)	0.23715 (16)	0.14335 (9)	0.0293 (3)	
H20	0.972915	0.140179	0.123847	0.035*	
C21	0.84900 (16)	0.28296 (14)	0.24402 (9)	0.0246 (2)	
H21	0.907679	0.218296	0.293053	0.030*	
C22_1	0.8462 (2)	0.2795 (2)	−0.03626 (11)	0.0428 (3)	0.53 (3)
F1_1	0.7831 (15)	0.3834 (12)	−0.1017 (5)	0.0620 (16)	0.53 (3)
F2_1	1.0337 (8)	0.2311 (12)	−0.0710 (7)	0.0480 (11)	0.53 (3)
F3_1	0.7692 (17)	0.1384 (13)	−0.0452 (5)	0.0641 (17)	0.53 (3)
C22_2	0.8462 (2)	0.2795 (2)	−0.03626 (11)	0.0428 (3)	0.47 (3)
F1_2	0.8272 (15)	0.4141 (10)	−0.0981 (6)	0.0622 (14)	0.47 (3)
F2_2	1.0216 (12)	0.2031 (17)	−0.0622 (8)	0.065 (2)	0.47 (3)
F3_2	0.7142 (14)	0.1977 (19)	−0.0581 (5)	0.0660 (18)	0.47 (3)

### 5,11-Diphenyl-6,12-bis[4-(trifluoromethyl)phenyl]tetracene (1) Atomic displacement parameters (Å^2^)

**Table:**

	*U*^11^	*U*^22^	*U*^33^	*U*^12^	*U*^13^	*U*^23^
C1	0.0213 (5)	0.0233 (5)	0.0225 (5)	−0.0027 (4)	−0.0010 (4)	−0.0039 (4)
C2	0.0217 (5)	0.0269 (5)	0.0280 (6)	−0.0050 (4)	−0.0003 (4)	−0.0026 (4)
C3	0.0247 (5)	0.0257 (5)	0.0286 (6)	−0.0089 (4)	−0.0054 (4)	−0.0022 (4)
C4	0.0256 (5)	0.0225 (5)	0.0215 (5)	−0.0066 (4)	−0.0053 (4)	−0.0025 (4)
C5	0.0212 (5)	0.0184 (4)	0.0185 (5)	−0.0034 (4)	−0.0056 (4)	−0.0018 (3)
C6	0.0205 (5)	0.0184 (4)	0.0163 (4)	−0.0025 (4)	−0.0048 (4)	−0.0028 (3)
C7	0.0183 (4)	0.0179 (4)	0.0165 (4)	−0.0020 (3)	−0.0037 (4)	−0.0027 (3)
C8	0.0199 (5)	0.0190 (4)	0.0161 (4)	−0.0012 (4)	−0.0030 (4)	−0.0033 (3)
C9	0.0195 (5)	0.0182 (4)	0.0183 (5)	−0.0015 (4)	−0.0032 (4)	−0.0017 (3)
C10	0.0213 (5)	0.0189 (4)	0.0191 (5)	−0.0044 (4)	−0.0027 (4)	−0.0041 (3)
C11	0.0220 (5)	0.0236 (5)	0.0210 (5)	−0.0021 (4)	−0.0056 (4)	−0.0038 (4)
C12	0.0279 (5)	0.0304 (6)	0.0216 (5)	−0.0057 (4)	−0.0065 (4)	−0.0069 (4)
C13	0.0380 (6)	0.0245 (5)	0.0275 (6)	−0.0054 (5)	−0.0036 (5)	−0.0110 (4)
C14	0.0394 (7)	0.0195 (5)	0.0320 (6)	0.0003 (5)	−0.0056 (5)	−0.0047 (4)
C15	0.0327 (6)	0.0209 (5)	0.0227 (5)	−0.0018 (4)	−0.0075 (4)	−0.0022 (4)
C16	0.0199 (5)	0.0225 (5)	0.0182 (5)	−0.0054 (4)	−0.0011 (4)	−0.0049 (4)
C17	0.0285 (5)	0.0235 (5)	0.0201 (5)	−0.0054 (4)	−0.0029 (4)	−0.0027 (4)
C18	0.0340 (6)	0.0330 (6)	0.0191 (5)	−0.0113 (5)	−0.0038 (4)	−0.0010 (4)
C19	0.0277 (6)	0.0404 (7)	0.0194 (5)	−0.0122 (5)	0.0017 (4)	−0.0107 (4)
C20	0.0227 (5)	0.0348 (6)	0.0295 (6)	−0.0020 (5)	−0.0001 (4)	−0.0154 (5)
C21	0.0220 (5)	0.0274 (5)	0.0244 (5)	−0.0014 (4)	−0.0040 (4)	−0.0076 (4)
C22_1	0.0404 (7)	0.0649 (10)	0.0243 (6)	−0.0168 (7)	0.0028 (5)	−0.0167 (6)
F1_1	0.067 (3)	0.094 (3)	0.0194 (11)	0.016 (2)	−0.0108 (16)	−0.0163 (16)
F2_1	0.0384 (15)	0.073 (3)	0.0275 (15)	−0.0050 (13)	0.0084 (11)	−0.0142 (15)
F3_1	0.078 (3)	0.082 (3)	0.0393 (17)	−0.041 (3)	0.0019 (18)	−0.0331 (19)
C22_2	0.0404 (7)	0.0649 (10)	0.0243 (6)	−0.0168 (7)	0.0028 (5)	−0.0167 (6)
F1_2	0.074 (3)	0.095 (2)	0.0162 (15)	−0.015 (2)	−0.0011 (16)	−0.0060 (15)
F2_2	0.069 (3)	0.080 (4)	0.036 (3)	0.003 (2)	0.0161 (19)	−0.032 (3)
F3_2	0.070 (3)	0.099 (5)	0.0381 (17)	−0.039 (3)	−0.0064 (17)	−0.032 (2)

### 5,11-Diphenyl-6,12-bis[4-(trifluoromethyl)phenyl]tetracene (1) Geometric parameters (Å, º)

**Table:**

C1—C2	1.3608 (16)	C13—C14	1.3849 (18)
C1—C9^i^	1.4369 (15)	C13—H13	0.9500
C1—H1	0.9500	C14—C15	1.3941 (16)
C2—C3	1.4205 (16)	C14—H14	0.9500
C2—H2	0.9500	C15—H15	0.9500
C3—C4	1.3610 (16)	C16—C17	1.3913 (15)
C3—H3	0.9500	C16—C21	1.3961 (15)
C4—C5	1.4348 (14)	C17—C18	1.3931 (15)
C4—H4	0.9500	C17—H17	0.9500
C5—C6	1.4033 (14)	C18—C19	1.3843 (18)
C5—C9^i^	1.4375 (14)	C18—H18	0.9500
C6—C7	1.4263 (14)	C19—C20	1.3873 (19)
C6—C10	1.4995 (14)	C19—C22_2	1.4985 (17)
C7—C8	1.4263 (14)	C19—C22_1	1.4985 (17)
C7—C7^i^	1.4703 (19)	C20—C21	1.3887 (16)
C8—C9	1.4023 (14)	C20—H20	0.9500
C8—C16	1.4999 (14)	C21—H21	0.9500
C10—C15	1.3934 (15)	C22_1—F1_1	1.304 (6)
C10—C11	1.3964 (15)	C22_1—F2_1	1.341 (6)
C11—C12	1.3913 (15)	C22_1—F3_1	1.391 (5)
C11—H11	0.9500	C22_2—F2_2	1.310 (7)
C12—C13	1.3859 (17)	C22_2—F3_2	1.312 (5)
C12—H12	0.9500	C22_2—F1_2	1.385 (7)
			
C2—C1—C9^i^	121.68 (10)	C13—C14—C15	120.01 (11)
C2—C1—H1	119.2	C13—C14—H14	120.0
C9^i^—C1—H1	119.2	C15—C14—H14	120.0
C1—C2—C3	120.25 (10)	C10—C15—C14	120.75 (10)
C1—C2—H2	119.9	C10—C15—H15	119.6
C3—C2—H2	119.9	C14—C15—H15	119.6
C4—C3—C2	120.08 (10)	C17—C16—C21	118.79 (10)
C4—C3—H3	120.0	C17—C16—C8	118.56 (9)
C2—C3—H3	120.0	C21—C16—C8	122.33 (10)
C3—C4—C5	121.85 (10)	C16—C17—C18	120.88 (11)
C3—C4—H4	119.1	C16—C17—H17	119.6
C5—C4—H4	119.1	C18—C17—H17	119.6
C6—C5—C4	121.80 (9)	C19—C18—C17	119.39 (11)
C6—C5—C9^i^	120.11 (9)	C19—C18—H18	120.3
C4—C5—C9^i^	118.03 (9)	C17—C18—H18	120.3
C5—C6—C7	120.62 (9)	C18—C19—C20	120.61 (11)
C5—C6—C10	115.56 (9)	C18—C19—C22_2	120.29 (12)
C7—C6—C10	123.53 (9)	C20—C19—C22_2	119.07 (12)
C6—C7—C8	122.23 (9)	C18—C19—C22_1	120.29 (12)
C6—C7—C7^i^	119.09 (11)	C20—C19—C22_1	119.07 (12)
C8—C7—C7^i^	118.69 (11)	C19—C20—C21	119.64 (11)
C9—C8—C7	120.80 (9)	C19—C20—H20	120.2
C9—C8—C16	115.15 (9)	C21—C20—H20	120.2
C7—C8—C16	123.75 (9)	C20—C21—C16	120.64 (11)
C8—C9—C1^i^	121.55 (9)	C20—C21—H21	119.7
C8—C9—C5^i^	120.27 (9)	C16—C21—H21	119.7
C1^i^—C9—C5^i^	118.08 (9)	F1_1—C22_1—F2_1	106.7 (5)
C15—C10—C11	118.58 (10)	F1_1—C22_1—F3_1	108.0 (3)
C15—C10—C6	118.84 (9)	F2_1—C22_1—F3_1	100.8 (5)
C11—C10—C6	122.37 (9)	F1_1—C22_1—C19	115.9 (4)
C12—C11—C10	120.59 (10)	F2_1—C22_1—C19	113.7 (4)
C12—C11—H11	119.7	F3_1—C22_1—C19	110.6 (3)
C10—C11—H11	119.7	F2_2—C22_2—F3_2	112.2 (6)
C13—C12—C11	120.20 (11)	F2_2—C22_2—F1_2	107.4 (6)
C13—C12—H12	119.9	F3_2—C22_2—F1_2	103.0 (4)
C11—C12—H12	119.9	F2_2—C22_2—C19	112.4 (5)
C14—C13—C12	119.84 (10)	F3_2—C22_2—C19	112.7 (3)
C14—C13—H13	120.1	F1_2—C22_2—C19	108.5 (4)
C12—C13—H13	120.1		
			
C9^i^—C1—C2—C3	0.79 (17)	C6—C10—C15—C14	176.95 (11)
C1—C2—C3—C4	0.87 (18)	C13—C14—C15—C10	−0.75 (19)
C2—C3—C4—C5	−1.44 (17)	C9—C8—C16—C17	−85.58 (12)
C3—C4—C5—C6	177.58 (10)	C7—C8—C16—C17	88.13 (13)
C3—C4—C5—C9^i^	0.34 (16)	C9—C8—C16—C21	87.83 (13)
C4—C5—C6—C7	177.15 (9)	C7—C8—C16—C21	−98.47 (13)
C9^i^—C5—C6—C7	−5.67 (15)	C21—C16—C17—C18	1.56 (17)
C4—C5—C6—C10	−8.83 (14)	C8—C16—C17—C18	175.21 (10)
C9^i^—C5—C6—C10	168.36 (9)	C16—C17—C18—C19	0.25 (18)
C5—C6—C7—C8	−174.59 (9)	C17—C18—C19—C20	−1.17 (18)
C10—C6—C7—C8	11.87 (15)	C17—C18—C19—C22_2	176.56 (11)
C5—C6—C7—C7^i^	5.51 (17)	C17—C18—C19—C22_1	176.56 (11)
C10—C6—C7—C7^i^	−168.03 (11)	C18—C19—C20—C21	0.23 (19)
C6—C7—C8—C9	−174.68 (9)	C22_2—C19—C20—C21	−177.52 (11)
C7^i^—C7—C8—C9	5.21 (17)	C22_1—C19—C20—C21	−177.52 (11)
C6—C7—C8—C16	11.95 (15)	C19—C20—C21—C16	1.63 (18)
C7^i^—C7—C8—C16	−168.15 (11)	C17—C16—C21—C20	−2.51 (17)
C7—C8—C9—C1^i^	178.61 (9)	C8—C16—C21—C20	−175.90 (10)
C16—C8—C9—C1^i^	−7.48 (14)	C18—C19—C22_1—F1_1	9.6 (7)
C7—C8—C9—C5^i^	−5.19 (15)	C20—C19—C22_1—F1_1	−172.7 (7)
C16—C8—C9—C5^i^	168.72 (9)	C18—C19—C22_1—F2_1	133.7 (5)
C5—C6—C10—C15	−88.19 (12)	C20—C19—C22_1—F2_1	−48.5 (5)
C7—C6—C10—C15	85.65 (13)	C18—C19—C22_1—F3_1	−113.8 (7)
C5—C6—C10—C11	86.42 (13)	C20—C19—C22_1—F3_1	64.0 (7)
C7—C6—C10—C11	−99.74 (13)	C18—C19—C22_2—F2_2	146.7 (8)
C15—C10—C11—C12	−2.11 (17)	C20—C19—C22_2—F2_2	−35.6 (8)
C6—C10—C11—C12	−176.74 (10)	C18—C19—C22_2—F3_2	−85.3 (9)
C10—C11—C12—C13	0.71 (18)	C20—C19—C22_2—F3_2	92.4 (9)
C11—C12—C13—C14	0.72 (19)	C18—C19—C22_2—F1_2	28.0 (5)
C12—C13—C14—C15	−0.7 (2)	C20—C19—C22_2—F1_2	−154.2 (5)
C11—C10—C15—C14	2.13 (18)		

Symmetry code: (i) −*x*+1, −*y*+1, −*z*+1.

### 5,11-Bis(4-tert-butylphenyl)-6,12-diphenyltetracene (2) Crystal data

**Table:**

C_50_H_44_	*F*(000) = 688
*M**_r_* = 644.85	*D*_x_ = 1.224 Mg m^−^^3^
Monoclinic, *P*2_1_/*c*	Cu *K*α radiation, λ = 1.54178 Å
*a* = 17.4565 (15) Å	Cell parameters from 9738 reflections
*b* = 7.2014 (6) Å	θ = 6.7–74.6°
*c* = 13.9356 (12) Å	µ = 0.52 mm^−^^1^
β = 92.593 (2)°	*T* = 100 K
*V* = 1750.1 (3) Å^3^	Block, red
*Z* = 2	0.50 × 0.25 × 0.10 mm

### 5,11-Bis(4-tert-butylphenyl)-6,12-diphenyltetracene (2) Data collection

**Table:**

Bruker Photon-II CMOS diffractometer	3431 reflections with *I* > 2σ(*I*)
φ and ω scans	*R*_int_ = 0.034
Absorption correction: multi-scan (SADABS; Krause *et al.*, 2015)	θ_max_ = 74.8°, θ_min_ = 5.1°
*T*_min_ = 0.620, *T*_max_ = 0.754	*h* = −21→21
34891 measured reflections	*k* = −9→8
3549 independent reflections	*l* = −17→17

### 5,11-Bis(4-tert-butylphenyl)-6,12-diphenyltetracene (2) Refinement

**Table:**

Refinement on *F*^2^	Primary atom site location: structure-invariant direct methods
Least-squares matrix: full	Secondary atom site location: difference Fourier map
*R*[*F*^2^ > 2σ(*F*^2^)] = 0.041	Hydrogen site location: inferred from neighbouring sites
*wR*(*F*^2^) = 0.105	H-atom parameters constrained
*S* = 1.08	*w* = 1/[σ^2^(*F*_o_^2^) + (0.0445*P*)^2^ + 0.7175*P*] where *P* = (*F*_o_^2^ + 2*F*_c_^2^)/3
3549 reflections	(Δ/σ)_max_ = 0.001
229 parameters	Δρ_max_ = 0.23 e Å^−^^3^
0 restraints	Δρ_min_ = −0.21 e Å^−^^3^

### 5,11-Bis(4-tert-butylphenyl)-6,12-diphenyltetracene (2) Special details

**Table:**

Geometry. All esds (except the esd in the dihedral angle between two l.s. planes) are estimated using the full covariance matrix. The cell esds are taken into account individually in the estimation of esds in distances, angles and torsion angles; correlations between esds in cell parameters are only used when they are defined by crystal symmetry. An approximate (isotropic) treatment of cell esds is used for estimating esds involving l.s. planes.

### 5,11-Bis(4-tert-butylphenyl)-6,12-diphenyltetracene (2) Fractional atomic coordinates and isotropic or equivalent isotropic displacement parameters (Å^2^)

**Table:**

	*x*	*y*	*z*	*U*_iso_*/*U*_eq_	
C1	0.54048 (6)	0.20094 (15)	0.41691 (7)	0.0182 (2)	
C2	0.58134 (6)	0.35267 (14)	0.45665 (7)	0.0181 (2)	
C3	0.54203 (6)	0.49972 (14)	0.50207 (7)	0.0175 (2)	
C4	0.58218 (6)	0.64679 (14)	0.55162 (7)	0.0180 (2)	
C5	0.54196 (6)	0.79852 (15)	0.58719 (7)	0.0181 (2)	
C7	0.54203 (6)	1.09596 (15)	0.67182 (8)	0.0226 (2)	
H7	0.569397	1.196553	0.701165	0.027*	
C6	0.58095 (6)	0.95208 (15)	0.63386 (7)	0.0209 (2)	
H6	0.635417	0.952965	0.638264	0.025*	
C9	0.42127 (6)	0.95278 (15)	0.62588 (7)	0.0208 (2)	
H9	0.366824	0.954238	0.624903	0.025*	
C8	0.46085 (6)	1.09637 (16)	0.66778 (8)	0.0227 (2)	
H8	0.433919	1.197268	0.694413	0.027*	
C10	0.66605 (6)	0.63804 (14)	0.58043 (8)	0.0195 (2)	
C11	0.72187 (6)	0.73773 (15)	0.53354 (8)	0.0230 (2)	
H11	0.708001	0.806390	0.477187	0.028*	
C12	0.79762 (6)	0.73705 (17)	0.56887 (9)	0.0279 (3)	
H12	0.835310	0.804172	0.536078	0.033*	
C13	0.81862 (6)	0.63895 (18)	0.65175 (9)	0.0304 (3)	
H13	0.870494	0.638778	0.675522	0.036*	
C14	0.76359 (7)	0.54137 (18)	0.69960 (9)	0.0291 (3)	
H14	0.777466	0.474931	0.756664	0.035*	
C15	0.68800 (6)	0.54111 (16)	0.66375 (8)	0.0237 (2)	
H15	0.650539	0.473505	0.696692	0.028*	
C16	0.66497 (6)	0.36066 (14)	0.43696 (8)	0.0193 (2)	
C17	0.68730 (6)	0.46152 (16)	0.35761 (8)	0.0232 (2)	
H17	0.650177	0.532606	0.321745	0.028*	
C18	0.76253 (7)	0.46026 (17)	0.32994 (8)	0.0267 (3)	
H18	0.776043	0.530974	0.275692	0.032*	
C19	0.81886 (6)	0.35704 (16)	0.38025 (9)	0.0259 (3)	
C20	0.79632 (6)	0.25727 (16)	0.46023 (9)	0.0263 (3)	
H20	0.833480	0.187196	0.496597	0.032*	
C21	0.72085 (6)	0.25820 (15)	0.48778 (8)	0.0230 (2)	
H21	0.707199	0.187879	0.542092	0.028*	
C22	0.90252 (7)	0.36360 (18)	0.35078 (11)	0.0349 (3)	
C23	0.90723 (8)	0.3565 (2)	0.24076 (12)	0.0470 (4)	
H23A	0.961087	0.361075	0.223849	0.071*	
H23B	0.879717	0.462884	0.212042	0.071*	
H23C	0.883899	0.241031	0.216370	0.071*	
C24	0.94952 (8)	0.2022 (2)	0.39435 (14)	0.0507 (4)	
H24A	1.001657	0.206593	0.371175	0.076*	
H24B	0.925342	0.084236	0.375207	0.076*	
H24C	0.951606	0.212450	0.464546	0.076*	
C25	0.93705 (7)	0.5489 (2)	0.38635 (12)	0.0426 (4)	
H25A	0.990449	0.557346	0.367906	0.064*	
H25B	0.935184	0.555463	0.456447	0.064*	
H25C	0.907536	0.651923	0.357381	0.064*	

### 5,11-Bis(4-tert-butylphenyl)-6,12-diphenyltetracene (2) Atomic displacement parameters (Å^2^)

**Table:**

	*U*^11^	*U*^22^	*U*^33^	*U*^12^	*U*^13^	*U*^23^
C1	0.0205 (5)	0.0192 (5)	0.0150 (5)	0.0013 (4)	0.0023 (4)	0.0020 (4)
C2	0.0189 (5)	0.0193 (5)	0.0161 (5)	0.0008 (4)	0.0010 (4)	0.0018 (4)
C3	0.0187 (5)	0.0182 (5)	0.0157 (5)	−0.0004 (4)	0.0019 (4)	0.0019 (4)
C4	0.0185 (5)	0.0193 (5)	0.0163 (5)	−0.0011 (4)	0.0025 (4)	0.0017 (4)
C5	0.0205 (5)	0.0192 (5)	0.0146 (5)	−0.0014 (4)	0.0018 (4)	0.0016 (4)
C7	0.0293 (6)	0.0193 (5)	0.0190 (5)	−0.0030 (4)	0.0008 (4)	−0.0021 (4)
C6	0.0225 (5)	0.0218 (5)	0.0186 (5)	−0.0029 (4)	0.0018 (4)	0.0000 (4)
C9	0.0224 (5)	0.0216 (5)	0.0185 (5)	0.0032 (4)	0.0019 (4)	0.0007 (4)
C8	0.0296 (6)	0.0193 (5)	0.0194 (5)	0.0038 (4)	0.0031 (4)	−0.0011 (4)
C10	0.0189 (5)	0.0186 (5)	0.0213 (5)	−0.0002 (4)	0.0019 (4)	−0.0039 (4)
C11	0.0225 (5)	0.0208 (5)	0.0258 (5)	−0.0011 (4)	0.0033 (4)	−0.0008 (4)
C12	0.0209 (5)	0.0262 (6)	0.0370 (6)	−0.0038 (4)	0.0062 (5)	−0.0039 (5)
C13	0.0183 (5)	0.0352 (7)	0.0372 (7)	0.0007 (5)	−0.0032 (5)	−0.0061 (5)
C14	0.0253 (6)	0.0354 (7)	0.0260 (6)	0.0042 (5)	−0.0029 (4)	0.0002 (5)
C15	0.0219 (5)	0.0267 (6)	0.0225 (5)	−0.0001 (4)	0.0026 (4)	−0.0006 (4)
C16	0.0186 (5)	0.0185 (5)	0.0209 (5)	0.0000 (4)	0.0024 (4)	−0.0041 (4)
C17	0.0229 (5)	0.0248 (6)	0.0220 (5)	0.0008 (4)	0.0012 (4)	0.0000 (4)
C18	0.0267 (6)	0.0287 (6)	0.0251 (6)	−0.0028 (5)	0.0078 (4)	0.0005 (5)
C19	0.0207 (5)	0.0240 (6)	0.0335 (6)	−0.0017 (4)	0.0069 (4)	−0.0061 (5)
C20	0.0202 (5)	0.0240 (6)	0.0346 (6)	0.0026 (4)	0.0004 (4)	−0.0003 (5)
C21	0.0218 (5)	0.0211 (5)	0.0260 (5)	0.0003 (4)	0.0025 (4)	0.0015 (4)
C22	0.0222 (6)	0.0322 (7)	0.0512 (8)	−0.0016 (5)	0.0119 (5)	−0.0045 (6)
C23	0.0356 (7)	0.0495 (9)	0.0582 (9)	−0.0060 (6)	0.0270 (7)	−0.0099 (7)
C24	0.0228 (6)	0.0454 (9)	0.0853 (12)	0.0066 (6)	0.0168 (7)	0.0058 (8)
C25	0.0235 (6)	0.0416 (8)	0.0635 (9)	−0.0067 (6)	0.0108 (6)	−0.0067 (7)

### 5,11-Bis(4-tert-butylphenyl)-6,12-diphenyltetracene (2) Geometric parameters (Å, º)

**Table:**

C1—C2	1.4049 (15)	C14—H14	0.9500
C1—C9^i^	1.4362 (15)	C15—H15	0.9500
C1—C5^i^	1.4378 (14)	C16—C21	1.3910 (15)
C2—C3	1.4256 (14)	C16—C17	1.3934 (15)
C2—C16	1.4986 (14)	C17—C18	1.3854 (15)
C3—C4	1.4304 (14)	C17—H17	0.9500
C3—C3^i^	1.4659 (19)	C18—C19	1.3959 (17)
C4—C5	1.4014 (15)	C18—H18	0.9500
C4—C10	1.5018 (14)	C19—C20	1.3975 (17)
C5—C6	1.4384 (15)	C19—C22	1.5356 (15)
C7—C6	1.3592 (16)	C20—C21	1.3888 (15)
C7—C8	1.4157 (16)	C20—H20	0.9500
C7—H7	0.9500	C21—H21	0.9500
C6—H6	0.9500	C22—C24	1.532 (2)
C9—C8	1.3605 (16)	C22—C25	1.5369 (19)
C9—H9	0.9500	C22—C23	1.540 (2)
C8—H8	0.9500	C23—H23A	0.9800
C10—C15	1.3935 (16)	C23—H23B	0.9800
C10—C11	1.3963 (15)	C23—H23C	0.9800
C11—C12	1.3900 (16)	C24—H24A	0.9800
C11—H11	0.9500	C24—H24B	0.9800
C12—C13	1.3890 (18)	C24—H24C	0.9800
C12—H12	0.9500	C25—H25A	0.9800
C13—C14	1.3854 (18)	C25—H25B	0.9800
C13—H13	0.9500	C25—H25C	0.9800
C14—C15	1.3897 (16)		
			
C2—C1—C9^i^	121.73 (9)	C10—C15—H15	119.3
C2—C1—C5^i^	120.20 (9)	C21—C16—C17	117.75 (10)
C9^i^—C1—C5^i^	117.96 (9)	C21—C16—C2	123.57 (10)
C1—C2—C3	120.45 (9)	C17—C16—C2	118.37 (9)
C1—C2—C16	116.05 (9)	C18—C17—C16	121.35 (10)
C3—C2—C16	123.04 (9)	C18—C17—H17	119.3
C2—C3—C4	121.94 (9)	C16—C17—H17	119.3
C2—C3—C3^i^	119.06 (11)	C17—C18—C19	121.25 (11)
C4—C3—C3^i^	118.99 (11)	C17—C18—H18	119.4
C5—C4—C3	120.37 (9)	C19—C18—H18	119.4
C5—C4—C10	115.85 (9)	C18—C19—C20	117.20 (10)
C3—C4—C10	123.26 (9)	C18—C19—C22	120.40 (11)
C4—C5—C1^i^	120.31 (9)	C20—C19—C22	122.32 (11)
C4—C5—C6	121.66 (9)	C21—C20—C19	121.52 (11)
C1^i^—C5—C6	117.92 (9)	C21—C20—H20	119.2
C6—C7—C8	120.21 (10)	C19—C20—H20	119.2
C6—C7—H7	119.9	C20—C21—C16	120.93 (10)
C8—C7—H7	119.9	C20—C21—H21	119.5
C7—C6—C5	121.83 (10)	C16—C21—H21	119.5
C7—C6—H6	119.1	C24—C22—C19	111.67 (11)
C5—C6—H6	119.1	C24—C22—C25	109.61 (12)
C8—C9—C1^i^	121.83 (10)	C19—C22—C25	107.72 (10)
C8—C9—H9	119.1	C24—C22—C23	108.52 (12)
C1^i^—C9—H9	119.1	C19—C22—C23	111.08 (11)
C9—C8—C7	120.23 (10)	C25—C22—C23	108.18 (12)
C9—C8—H8	119.9	C22—C23—H23A	109.5
C7—C8—H8	119.9	C22—C23—H23B	109.5
C15—C10—C11	118.43 (10)	H23A—C23—H23B	109.5
C15—C10—C4	118.15 (9)	C22—C23—H23C	109.5
C11—C10—C4	123.08 (10)	H23A—C23—H23C	109.5
C12—C11—C10	120.35 (11)	H23B—C23—H23C	109.5
C12—C11—H11	119.8	C22—C24—H24A	109.5
C10—C11—H11	119.8	C22—C24—H24B	109.5
C13—C12—C11	120.49 (11)	H24A—C24—H24B	109.5
C13—C12—H12	119.8	C22—C24—H24C	109.5
C11—C12—H12	119.8	H24A—C24—H24C	109.5
C14—C13—C12	119.71 (11)	H24B—C24—H24C	109.5
C14—C13—H13	120.1	C22—C25—H25A	109.5
C12—C13—H13	120.1	C22—C25—H25B	109.5
C13—C14—C15	119.70 (11)	H25A—C25—H25B	109.5
C13—C14—H14	120.1	C22—C25—H25C	109.5
C15—C14—H14	120.1	H25A—C25—H25C	109.5
C14—C15—C10	121.31 (11)	H25B—C25—H25C	109.5
C14—C15—H15	119.3		
			
C9^i^—C1—C2—C3	−177.47 (9)	C10—C11—C12—C13	−0.69 (17)
C5^i^—C1—C2—C3	6.42 (15)	C11—C12—C13—C14	−0.11 (18)
C9^i^—C1—C2—C16	10.12 (14)	C12—C13—C14—C15	0.59 (18)
C5^i^—C1—C2—C16	−165.98 (9)	C13—C14—C15—C10	−0.28 (18)
C1—C2—C3—C4	173.65 (9)	C11—C10—C15—C14	−0.51 (16)
C16—C2—C3—C4	−14.50 (15)	C4—C10—C15—C14	−174.03 (10)
C1—C2—C3—C3^i^	−6.33 (17)	C1—C2—C16—C21	−80.56 (13)
C16—C2—C3—C3^i^	165.53 (11)	C3—C2—C16—C21	107.25 (12)
C2—C3—C4—C5	173.66 (9)	C1—C2—C16—C17	92.84 (12)
C3^i^—C3—C4—C5	−6.37 (17)	C3—C2—C16—C17	−79.35 (13)
C2—C3—C4—C10	−14.88 (15)	C21—C16—C17—C18	0.01 (16)
C3^i^—C3—C4—C10	165.09 (11)	C2—C16—C17—C18	−173.78 (10)
C3—C4—C5—C1^i^	6.43 (15)	C16—C17—C18—C19	0.32 (18)
C10—C4—C5—C1^i^	−165.64 (9)	C17—C18—C19—C20	−0.80 (17)
C3—C4—C5—C6	−177.43 (9)	C17—C18—C19—C22	−177.59 (11)
C10—C4—C5—C6	10.50 (14)	C18—C19—C20—C21	0.98 (17)
C8—C7—C6—C5	1.16 (16)	C22—C19—C20—C21	177.71 (11)
C4—C5—C6—C7	−177.35 (10)	C19—C20—C21—C16	−0.68 (18)
C1^i^—C5—C6—C7	−1.12 (15)	C17—C16—C21—C20	0.16 (16)
C1^i^—C9—C8—C7	−1.19 (16)	C2—C16—C21—C20	173.60 (10)
C6—C7—C8—C9	0.01 (16)	C18—C19—C22—C24	−163.48 (12)
C5—C4—C10—C15	90.96 (12)	C20—C19—C22—C24	19.89 (18)
C3—C4—C10—C15	−80.85 (13)	C18—C19—C22—C25	76.12 (15)
C5—C4—C10—C11	−82.23 (13)	C20—C19—C22—C25	−100.51 (14)
C3—C4—C10—C11	105.95 (12)	C18—C19—C22—C23	−42.19 (16)
C15—C10—C11—C12	0.99 (16)	C20—C19—C22—C23	141.18 (13)
C4—C10—C11—C12	174.17 (10)		

Symmetry code: (i) −*x*+1, −*y*+1, −*z*+1.

### (CIYXUF) Crystal data

**Table:**

C_44_H_26_F_6_	*F*(000) = 688
*M**_r_* = 668.68	Mo *K*α radiation, λ = 0.71075 Å
Monoclinic, *P*2_1_/*c*	Cell parameters from 8235 reflections
*a* = 15.9782 (6) Å	θ = 1.8–27.5°
*b* = 7.2762 (2) Å	µ = 0.11 mm^−^^1^
*c* = 13.9814 (6) Å	*T* = 123 K
β = 102.701 (2)°	Prism, orange
*V* = 1585.71 (10) Å^3^	0.35 × 0.28 × 0.10 mm
*Z* = 2	

### (CIYXUF) Data collection

**Table:**

Rigaku RAXIS II diffractometer	3643 independent reflections
Radiation source: fine-focus sealed tube	3222 reflections with *I* > 2σ(*I*)
Graphite Monochromator monochromator	*R*_int_ = 0.051
Detector resolution: 10.0000 pixels mm^-1^	θ_max_ = 27.5°, θ_min_ = 3.0°
profile data from ω–scans	*h* = −20→20
Absorption correction: numerical (*CrystalClear-SM Expert*; Rigaku, 2009)'	*k* = −9→9
*T*_min_ = 0.971, *T*_max_ = 0.992	*l* = −18→18
15137 measured reflections	

### (CIYXUF) Refinement

**Table:**

Refinement on *F*^2^	Hydrogen site location: inferred from neighbouring sites
Least-squares matrix: full	H-atom parameters constrained
*R*[*F*^2^ > 2σ(*F*^2^)] = 0.066	*w* = 1/[σ^2^(*F*_o_^2^) + (0.0244*P*)^2^ + 1.4827*P*] where *P* = (*F*_o_^2^ + 2*F*_c_^2^)/3
*wR*(*F*^2^) = 0.135	(Δ/σ)_max_ < 0.001
*S* = 1.11	Δρ_max_ = 0.37 e Å^−^^3^
3643 reflections	Δρ_min_ = −0.30 e Å^−^^3^
197 parameters	Extinction correction: SHELXL, Fc^*^=kFc[1+0.001xFc^2^λ^3^/sin(2θ)]^-1/4^
183 restraints	Extinction coefficient: 0.0048 (11)

### (CIYXUF) Special details

**Table:**

Geometry. All esds (except the esd in the dihedral angle between two l.s. planes) are estimated using the full covariance matrix. The cell esds are taken into account individually in the estimation of esds in distances, angles and torsion angles; correlations between esds in cell parameters are only used when they are defined by crystal symmetry. An approximate (isotropic) treatment of cell esds is used for estimating esds involving l.s. planes.
Refinement. 'Refinement of F^2^ against ALL reflections. The weighted R-factor wR and goodness of fit S are based on F^2^, conventional R-factors R are based on F, with F set to zero for negative F^2^. The threshold expression of F^2^ > 2sigma(F^2^) is used only for calculating R-factors(gt) etc. and is not relevant to the choice of reflections for refinement. R-factors based on F^2^ are statistically about twice as large as those based on F, and R- factors based on ALL data will be even larger.'

### (CIYXUF) Fractional atomic coordinates and isotropic or equivalent isotropic displacement parameters (Å^2^)

**Table:**

	*x*	*y*	*z*	*U*_iso_*/*U*_eq_	
C1	0.18597 (5)	−0.14438 (12)	0.97899 (6)	0.0226 (4)	
C2	0.24601 (6)	−0.24679 (9)	1.04470 (6)	0.0284 (5)	
H2	0.2288	−0.3170	1.0925	0.034*	
C3	0.33174 (6)	−0.24421 (11)	1.03891 (7)	0.0359 (5)	
H3	0.3719	−0.3127	1.0829	0.043*	
C4	0.35743 (6)	−0.13922 (15)	0.96741 (7)	0.0390 (6)	
H4	0.4148	−0.1375	0.9635	0.047*	
C5	0.29740 (7)	−0.03680 (15)	0.90170 (6)	0.0361 (5)	
H5	0.3146	0.0335	0.8539	0.043*	
C6	0.21167 (6)	−0.03938 (14)	0.90749 (5)	0.0280 (4)	
H6	0.1715	0.0291	0.8635	0.034*	
C7	0.18603 (5)	0.12510 (12)	1.12563 (6)	0.0221 (4)	
C8	0.20497 (6)	0.02091 (13)	1.21099 (6)	0.0260 (4)	
H8	0.1618	−0.0446	1.2309	0.031*	
C9	0.28849 (7)	0.01456 (14)	1.26666 (5)	0.0301 (5)	
H9	0.3012	−0.0552	1.3238	0.036*	
C10	0.35306 (5)	0.11241 (14)	1.23697 (6)	0.0309 (5)	
C11	0.33412 (6)	0.21660 (11)	1.15161 (7)	0.0304 (5)	
H11	0.3773	0.2821	1.1317	0.036*	
C12	0.25060 (6)	0.22295 (8)	1.09593 (6)	0.0261 (4)	
H12	0.2379	0.2927	1.0388	0.031*	
C13	0.09073 (11)	−0.1505 (3)	0.97772 (14)	0.0211 (4)	
C14	0.04716 (11)	−0.0020 (3)	1.01322 (13)	0.0200 (4)	
C15	0.09214 (11)	0.1403 (3)	1.07337 (14)	0.0202 (4)	
C16	0.04793 (12)	0.2929 (3)	1.09877 (14)	0.0209 (4)	
C17	0.09151 (13)	0.4412 (3)	1.15732 (14)	0.0240 (4)	
H17	0.1510	0.4380	1.1771	0.029*	
C18	0.04821 (13)	0.5857 (3)	1.18443 (15)	0.0269 (4)	
H18	0.0783	0.6800	1.2218	0.032*	
C19	−0.04255 (13)	0.5939 (3)	1.15618 (15)	0.0270 (4)	
H19	−0.0717	0.6945	1.1739	0.032*	
C20	−0.08696 (13)	0.4547 (3)	1.10322 (14)	0.0246 (4)	
H20	−0.1465	0.4604	1.0868	0.029*	
C21	0.04465 (12)	−0.2987 (3)	0.92823 (14)	0.0211 (4)	
C22	0.44252 (14)	0.1128 (3)	1.2993 (2)	0.0402 (6)	
F1	0.46000 (10)	−0.0327 (3)	1.35651 (16)	0.0779 (7)	
F2	0.50261 (9)	0.1150 (3)	1.24718 (15)	0.0735 (6)	
F3	0.45791 (10)	0.2595 (3)	1.35632 (16)	0.0861 (8)	

### (CIYXUF) Atomic displacement parameters (Å^2^)

**Table:**

	*U*^11^	*U*^22^	*U*^33^	*U*^12^	*U*^13^	*U*^23^
C1	0.0224 (9)	0.0223 (9)	0.0215 (9)	−0.0004 (7)	0.0013 (7)	−0.0045 (7)
C2	0.0249 (10)	0.0268 (10)	0.0307 (11)	0.0025 (8)	0.0001 (8)	−0.0005 (8)
C3	0.0244 (10)	0.0347 (11)	0.0437 (13)	0.0060 (9)	−0.0031 (9)	−0.0040 (10)
C4	0.0217 (10)	0.0492 (14)	0.0459 (14)	−0.0021 (10)	0.0072 (9)	−0.0117 (11)
C5	0.0308 (11)	0.0441 (13)	0.0352 (12)	−0.0078 (10)	0.0111 (9)	−0.0052 (10)
C6	0.0269 (10)	0.0318 (11)	0.0240 (10)	−0.0007 (8)	0.0032 (8)	−0.0011 (8)
C7	0.0208 (9)	0.0212 (9)	0.0224 (9)	−0.0011 (7)	0.0006 (7)	−0.0040 (7)
C8	0.0242 (9)	0.0285 (10)	0.0240 (10)	−0.0018 (8)	0.0022 (8)	0.0001 (8)
C9	0.0288 (10)	0.0325 (11)	0.0248 (11)	0.0018 (9)	−0.0031 (8)	−0.0004 (8)
C10	0.0222 (9)	0.0309 (11)	0.0350 (12)	0.0010 (8)	−0.0036 (8)	−0.0057 (9)
C11	0.0231 (9)	0.0270 (10)	0.0404 (12)	−0.0044 (8)	0.0057 (9)	−0.0028 (9)
C12	0.0239 (9)	0.0252 (10)	0.0277 (10)	−0.0017 (8)	0.0027 (8)	0.0012 (8)
C13	0.0210 (9)	0.0218 (9)	0.0186 (9)	0.0003 (7)	0.0002 (7)	0.0018 (7)
C14	0.0214 (9)	0.0207 (9)	0.0172 (9)	−0.0010 (7)	0.0026 (7)	0.0019 (7)
C15	0.0202 (8)	0.0212 (9)	0.0183 (9)	−0.0009 (7)	0.0024 (7)	0.0014 (7)
C16	0.0240 (9)	0.0201 (9)	0.0174 (9)	−0.0019 (7)	0.0021 (7)	0.0021 (7)
C17	0.0264 (9)	0.0238 (9)	0.0210 (9)	−0.0044 (8)	0.0038 (8)	−0.0006 (8)
C18	0.0339 (10)	0.0225 (9)	0.0234 (10)	−0.0043 (8)	0.0040 (8)	−0.0026 (8)
C19	0.0344 (10)	0.0223 (9)	0.0245 (10)	0.0032 (8)	0.0068 (8)	−0.0004 (8)
C20	0.0253 (9)	0.0247 (9)	0.0226 (10)	0.0027 (8)	0.0029 (8)	−0.0003 (8)
C21	0.0218 (9)	0.0220 (9)	0.0183 (9)	0.0006 (7)	0.0019 (7)	0.0016 (7)
C22	0.0262 (11)	0.0402 (13)	0.0482 (14)	0.0014 (9)	−0.0046 (10)	−0.0037 (11)
F1	0.0375 (8)	0.0834 (13)	0.0952 (15)	−0.0024 (9)	−0.0234 (9)	0.0399 (11)
F2	0.0228 (7)	0.1234 (17)	0.0705 (12)	0.0027 (9)	0.0016 (7)	0.0002 (12)
F3	0.0403 (9)	0.0859 (13)	0.1087 (16)	0.0159 (9)	−0.0341 (9)	−0.0592 (12)

### (CIYXUF) Geometric parameters (Å, º)

**Table:**

C1—C2	1.3900	C13—C14	1.432 (3)
C1—C6	1.3900	C14—C15	1.425 (3)
C1—C13	1.5186 (19)	C14—C14^i^	1.471 (3)
C2—C3	1.3900	C15—C16	1.403 (3)
C3—C4	1.3900	C16—C17	1.438 (3)
C4—C5	1.3900	C16—C21^i^	1.445 (3)
C5—C6	1.3900	C17—C18	1.358 (3)
C7—C8	1.3900	C18—C19	1.418 (3)
C7—C12	1.3900	C19—C20	1.359 (3)
C7—C15	1.5213 (19)	C20—C21^i^	1.438 (3)
C8—C9	1.3900	C21—C20^i^	1.438 (3)
C9—C10	1.3900	C21—C16^i^	1.445 (3)
C10—C11	1.3900	C22—F1	1.320 (3)
C10—C22	1.501 (2)	C22—F3	1.323 (3)
C11—C12	1.3900	C22—F2	1.327 (3)
C13—C21	1.400 (3)		
			
C2—C1—C6	120.0	C15—C14—C14^i^	119.3 (2)
C2—C1—C13	122.43 (9)	C13—C14—C14^i^	118.5 (2)
C6—C1—C13	117.47 (9)	C16—C15—C14	120.52 (16)
C1—C2—C3	120.0	C16—C15—C7	115.72 (15)
C4—C3—C2	120.0	C14—C15—C7	123.16 (15)
C5—C4—C3	120.0	C15—C16—C17	122.02 (17)
C4—C5—C6	120.0	C15—C16—C21^i^	120.05 (17)
C5—C6—C1	120.0	C17—C16—C21^i^	117.80 (17)
C8—C7—C12	120.0	C18—C17—C16	121.82 (18)
C8—C7—C15	117.22 (9)	C17—C18—C19	120.50 (19)
C12—C7—C15	122.53 (9)	C20—C19—C18	120.00 (19)
C9—C8—C7	120.0	C19—C20—C21^i^	122.04 (18)
C8—C9—C10	120.0	C13—C21—C20^i^	121.83 (17)
C11—C10—C9	120.0	C13—C21—C16^i^	120.23 (17)
C11—C10—C22	119.80 (12)	C20^i^—C21—C16^i^	117.80 (17)
C9—C10—C22	120.14 (12)	F1—C22—F3	107.2 (2)
C12—C11—C10	120.0	F1—C22—F2	105.1 (2)
C11—C12—C7	120.0	F3—C22—F2	105.1 (2)
C21—C13—C14	120.63 (16)	F1—C22—C10	113.44 (19)
C21—C13—C1	116.43 (15)	F3—C22—C10	112.24 (18)
C14—C13—C1	122.43 (15)	F2—C22—C10	113.1 (2)
C15—C14—C13	122.16 (16)		

Symmetry code: (i) −*x*, −*y*, −*z*+2.

### 5,11-Bis(4-tert-butylphenyl)-6,12-diphenylnaphthacene (PIFHOC) Crystal data

**Table:**

C_50_H_44_	*F*(000) = 1376
*M**_r_* = 644.85	*D*_x_ = 1.141 Mg m^−^^3^
Monoclinic, *P*2_1_/*c*	Mo *K*α radiation, λ = 0.71073 Å
Hall symbol: -P 2ybc	Cell parameters from 3136 reflections
*a* = 23.527 (3) Å	θ = 3.0–25.0°
*b* = 9.0277 (10) Å	µ = 0.06 mm^−^^1^
*c* = 17.764 (2) Å	*T* = 292 K
β = 95.928 (4)°	Plate, translucent orange
*V* = 3752.8 (8) Å^3^	0.36 × 0.16 × 0.04 mm
*Z* = 4	

### 5,11-Bis(4-tert-butylphenyl)-6,12-diphenylnaphthacene (PIFHOC) Data collection

**Table:**

Brucker SMART CCD diffractometer	6626 independent reflections
Radiation source: fine-focus sealed tube	3478 reflections with *I* > 2σ(*I*)
Graphite monochromator	*R*_int_ = 0.100
φ and ω scans	θ_max_ = 25.0°, θ_min_ = 0.9°
Absorption correction: multi-scan (SADABS; Krause *et al.*, 2015)	*h* = −27→27
*T*_min_ = 0.990, *T*_max_ = 0.997	*k* = −10→10
31129 measured reflections	*l* = −21→21

### 5,11-Bis(4-tert-butylphenyl)-6,12-diphenylnaphthacene (PIFHOC) Refinement

**Table:**

Refinement on *F*^2^	Secondary atom site location: difference Fourier map
Least-squares matrix: full	Hydrogen site location: difmap and geom
*R*[*F*^2^ > 2σ(*F*^2^)] = 0.098	H atoms treated by a mixture of independent and constrained refinement
*wR*(*F*^2^) = 0.169	*w* = 1/[σ^2^(*F*_o_^2^) + (0.0241*P*)^2^ + 4.9951*P*] where *P* = (*F*_o_^2^ + 2*F*_c_^2^)/3
*S* = 1.11	(Δ/σ)_max_ = 0.004
6626 reflections	Δρ_max_ = 0.29 e Å^−^^3^
536 parameters	Δρ_min_ = −0.21 e Å^−^^3^
0 restraints	Extinction correction: SHELXL97, Fc^*^=kFc[1+0.001xFc^2^λ^3^/sin(2θ)]^-1/4^
Primary atom site location: structure-invariant direct methods	Extinction coefficient: 0.0020 (3)

### 5,11-Bis(4-tert-butylphenyl)-6,12-diphenylnaphthacene (PIFHOC) Special details

**Table:**

Geometry. All esds (except the esd in the dihedral angle between two l.s. planes) are estimated using the full covariance matrix. The cell esds are taken into account individually in the estimation of esds in distances, angles and torsion angles; correlations between esds in cell parameters are only used when they are defined by crystal symmetry. An approximate (isotropic) treatment of cell esds is used for estimating esds involving l.s. planes.
Refinement. Refinement of F^2^ against ALL reflections. The weighted R-factor wR and goodness of fit S are based on F^2^, conventional R-factors R are based on F, with F set to zero for negative F^2^. The threshold expression of F^2^ > 2sigma(F^2^) is used only for calculating R-factors(gt) etc. and is not relevant to the choice of reflections for refinement. R-factors based on F^2^ are statistically about twice as large as those based on F, and R- factors based on ALL data will be even larger.

### 5,11-Bis(4-tert-butylphenyl)-6,12-diphenylnaphthacene (PIFHOC) Fractional atomic coordinates and isotropic or equivalent isotropic displacement parameters (Å^2^)

**Table:**

	*x*	*y*	*z*	*U*_iso_*/*U*_eq_	
C1	0.4082 (2)	0.7231 (5)	0.3755 (3)	0.0571 (13)	
C2	0.43973 (19)	0.6785 (5)	0.4434 (3)	0.0544 (13)	
C3	0.35092 (18)	0.7072 (5)	0.3654 (2)	0.0444 (11)	
C4	0.41355 (18)	0.6099 (5)	0.4982 (3)	0.0481 (12)	
C5	0.32005 (16)	0.6423 (4)	0.4231 (2)	0.0388 (10)	
C6	0.35296 (15)	0.5814 (4)	0.4884 (2)	0.0352 (10)	
C7	0.26002 (16)	0.6412 (4)	0.4184 (2)	0.0378 (10)	
C8	0.32605 (15)	0.4935 (4)	0.5393 (2)	0.0356 (10)	
C9	0.23227 (15)	0.5692 (4)	0.4762 (2)	0.0350 (10)	
C10	0.26556 (15)	0.4713 (4)	0.5276 (2)	0.0357 (10)	
C11	0.17319 (15)	0.5862 (4)	0.4856 (2)	0.0353 (10)	
C12	0.23732 (16)	0.3584 (4)	0.5653 (2)	0.0370 (10)	
C13	0.14684 (15)	0.4909 (4)	0.5329 (2)	0.0360 (10)	
C14	0.17806 (16)	0.3674 (4)	0.5681 (2)	0.0387 (10)	
C15	0.08776 (17)	0.5078 (5)	0.5463 (2)	0.0464 (11)	
C16	0.14702 (19)	0.2603 (5)	0.6065 (3)	0.0512 (12)	
C17	0.0615 (2)	0.4075 (6)	0.5867 (3)	0.0550 (13)	
C18	0.0911 (2)	0.2800 (6)	0.6153 (3)	0.0604 (14)	
C20	0.22674 (17)	0.6982 (5)	0.3482 (2)	0.0483 (11)	
C21	0.2261 (2)	0.8465 (6)	0.3280 (3)	0.0723 (16)	
C22	0.1970 (3)	0.8925 (10)	0.2595 (5)	0.106 (3)	
C23	0.1690 (3)	0.7895 (13)	0.2123 (4)	0.120 (4)	
C24	0.1700 (3)	0.6451 (11)	0.2308 (3)	0.105 (3)	
C25	0.1988 (2)	0.5961 (7)	0.2987 (3)	0.0669 (15)	
C30	0.35873 (15)	0.4391 (4)	0.6107 (2)	0.0375 (10)	
C31	0.40131 (16)	0.3323 (5)	0.6126 (2)	0.0444 (11)	
C32	0.42905 (18)	0.2851 (5)	0.6807 (3)	0.0487 (12)	
C33	0.41595 (18)	0.3416 (5)	0.7495 (2)	0.0488 (11)	
C34	0.3746 (2)	0.4500 (5)	0.7468 (2)	0.0519 (12)	
C35	0.34595 (19)	0.4986 (5)	0.6788 (2)	0.0486 (12)	
C36	0.4456 (2)	0.2853 (6)	0.8254 (3)	0.0669 (14)	
C37	0.4903 (4)	0.3883 (8)	0.8554 (4)	0.202 (5)	
H37A	0.5054	0.3571	0.9052	0.303*	
H37B	0.4745	0.4860	0.8580	0.303*	
H37C	0.5204	0.3893	0.8228	0.303*	
C38	0.4708 (3)	0.1331 (7)	0.8175 (3)	0.146 (3)	
H38A	0.5047	0.1407	0.7920	0.219*	
H38B	0.4435	0.0710	0.7886	0.219*	
H38C	0.4802	0.0908	0.8667	0.219*	
C39	0.4024 (4)	0.2652 (12)	0.8809 (4)	0.218 (6)	
H39A	0.4184	0.2046	0.9222	0.327*	
H39B	0.3689	0.2180	0.8563	0.327*	
H39C	0.3922	0.3601	0.8999	0.327*	
C40	0.14101 (16)	0.7183 (4)	0.4525 (2)	0.0371 (10)	
C41	0.15368 (19)	0.8579 (5)	0.4817 (2)	0.0491 (12)	
C42	0.1243 (2)	0.9821 (5)	0.4531 (3)	0.0579 (13)	
C43	0.08086 (18)	0.9718 (5)	0.3945 (2)	0.0446 (11)	
C44	0.06779 (18)	0.8326 (5)	0.3667 (2)	0.0490 (12)	
C45	0.09662 (17)	0.7067 (5)	0.3952 (2)	0.0437 (11)	
C46	0.0491 (2)	1.1098 (5)	0.3632 (3)	0.0653 (14)	
C47	0.0073 (3)	1.1571 (8)	0.4184 (4)	0.166 (4)	
H47A	0.0282	1.1912	0.4645	0.249*	
H47B	−0.0161	1.0743	0.4292	0.249*	
H47C	−0.0164	1.2357	0.3965	0.249*	
C48	0.0899 (3)	1.2343 (6)	0.3525 (4)	0.147 (3)	
H48A	0.1074	1.2666	0.4009	0.220*	
H48B	0.0695	1.3154	0.3273	0.220*	
H48C	0.1190	1.2005	0.3223	0.220*	
C49	0.0140 (3)	1.0800 (6)	0.2874 (3)	0.110 (2)	
H49A	−0.0043	1.1698	0.2690	0.165*	
H49B	−0.0145	1.0063	0.2941	0.165*	
H49C	0.0388	1.0450	0.2515	0.165*	
C50	0.26843 (17)	0.2211 (5)	0.5919 (3)	0.0473 (11)	
C51	0.2768 (2)	0.1763 (6)	0.6668 (3)	0.0656 (15)	
C52	0.3053 (2)	0.0437 (8)	0.6856 (4)	0.089 (2)	
C53	0.3249 (3)	−0.0439 (7)	0.6305 (6)	0.103 (3)	
C54	0.3169 (3)	−0.0008 (7)	0.5560 (5)	0.092 (2)	
C55	0.2890 (2)	0.1303 (5)	0.5368 (3)	0.0639 (14)	
H1	0.4291 (19)	0.759 (5)	0.335 (2)	0.077*	
H2	0.4827 (19)	0.693 (5)	0.454 (2)	0.077*	
H3	0.3284 (18)	0.744 (5)	0.317 (2)	0.077*	
H4	0.4347 (18)	0.574 (5)	0.547 (2)	0.077*	
H15	0.0685 (18)	0.600 (5)	0.524 (2)	0.077*	
H16	0.1680 (18)	0.174 (5)	0.630 (2)	0.077*	
H17	0.0193 (19)	0.428 (5)	0.596 (2)	0.077*	
H18	0.0713 (19)	0.218 (5)	0.644 (2)	0.077*	
H21	0.245 (2)	0.918 (6)	0.366 (3)	0.096*	
H22	0.197 (2)	0.993 (6)	0.249 (3)	0.096*	
H23	0.148 (2)	0.818 (6)	0.163 (3)	0.096*	
H24	0.152 (2)	0.564 (6)	0.199 (3)	0.096*	
H25	0.201 (2)	0.488 (6)	0.316 (3)	0.096*	
H31	0.4121 (18)	0.287 (5)	0.565 (2)	0.077*	
H32	0.4602 (19)	0.215 (5)	0.678 (2)	0.077*	
H34	0.3639 (18)	0.496 (5)	0.793 (2)	0.077*	
H35	0.3160 (18)	0.572 (5)	0.681 (2)	0.077*	
H41	0.1844 (18)	0.866 (5)	0.523 (2)	0.077*	
H42	0.1385 (18)	1.075 (5)	0.476 (2)	0.077*	
H44	0.0400 (18)	0.817 (5)	0.325 (2)	0.077*	
H45	0.0856 (18)	0.607 (5)	0.373 (2)	0.077*	
H51	0.263 (2)	0.241 (6)	0.706 (3)	0.096*	
H52	0.310 (2)	0.018 (6)	0.736 (3)	0.096*	
H53	0.346 (2)	−0.140 (6)	0.646 (3)	0.096*	
H54	0.329 (2)	−0.069 (6)	0.514 (3)	0.096*	
H55	0.281 (2)	0.163 (5)	0.480 (3)	0.096*	

### 5,11-Bis(4-tert-butylphenyl)-6,12-diphenylnaphthacene (PIFHOC) Atomic displacement parameters (Å^2^)

**Table:**

	*U*^11^	*U*^22^	*U*^33^	*U*^12^	*U*^13^	*U*^23^
C1	0.047 (3)	0.065 (3)	0.061 (3)	−0.008 (3)	0.018 (2)	0.010 (3)
C2	0.034 (3)	0.070 (3)	0.058 (3)	−0.011 (3)	0.000 (2)	0.009 (3)
C3	0.043 (3)	0.045 (3)	0.046 (3)	−0.002 (2)	0.009 (2)	0.008 (2)
C4	0.039 (3)	0.056 (3)	0.048 (3)	−0.004 (2)	−0.003 (2)	0.004 (2)
C5	0.039 (2)	0.034 (2)	0.043 (2)	−0.002 (2)	0.0004 (19)	0.0016 (19)
C6	0.028 (2)	0.035 (2)	0.042 (2)	−0.0030 (19)	0.0011 (18)	0.0019 (19)
C7	0.038 (2)	0.038 (2)	0.037 (2)	0.003 (2)	−0.0016 (18)	0.0045 (19)
C8	0.033 (2)	0.034 (2)	0.039 (2)	0.0025 (19)	0.0022 (18)	−0.0026 (19)
C9	0.032 (2)	0.036 (2)	0.036 (2)	0.0012 (19)	−0.0014 (18)	0.0010 (19)
C10	0.031 (2)	0.040 (2)	0.036 (2)	0.0034 (19)	0.0023 (17)	0.0007 (19)
C11	0.031 (2)	0.038 (2)	0.035 (2)	0.0015 (19)	−0.0029 (18)	0.0006 (19)
C12	0.035 (2)	0.040 (3)	0.037 (2)	0.004 (2)	0.0057 (18)	0.0036 (19)
C13	0.030 (2)	0.045 (3)	0.032 (2)	−0.001 (2)	0.0005 (17)	−0.005 (2)
C14	0.039 (2)	0.039 (3)	0.039 (2)	−0.004 (2)	0.0082 (19)	0.002 (2)
C15	0.038 (3)	0.057 (3)	0.045 (3)	−0.001 (2)	0.006 (2)	−0.001 (2)
C16	0.043 (3)	0.056 (3)	0.055 (3)	−0.005 (2)	0.004 (2)	0.008 (2)
C17	0.039 (3)	0.068 (3)	0.059 (3)	0.000 (3)	0.013 (2)	0.004 (3)
C18	0.049 (3)	0.072 (4)	0.063 (3)	−0.011 (3)	0.019 (2)	0.011 (3)
C20	0.041 (3)	0.065 (3)	0.041 (3)	0.010 (2)	0.010 (2)	0.012 (2)
C21	0.059 (3)	0.081 (4)	0.078 (4)	0.012 (3)	0.010 (3)	0.041 (3)
C22	0.079 (5)	0.127 (7)	0.115 (6)	0.033 (5)	0.028 (4)	0.076 (6)
C23	0.094 (6)	0.208 (11)	0.059 (5)	0.061 (7)	0.009 (4)	0.045 (6)
C24	0.088 (5)	0.168 (8)	0.053 (4)	0.050 (5)	−0.019 (3)	−0.023 (4)
C25	0.056 (3)	0.097 (4)	0.046 (3)	0.023 (3)	−0.003 (2)	−0.010 (3)
C30	0.027 (2)	0.043 (3)	0.043 (3)	−0.001 (2)	0.0014 (18)	0.004 (2)
C31	0.031 (2)	0.059 (3)	0.043 (3)	0.009 (2)	0.002 (2)	0.004 (2)
C32	0.037 (3)	0.056 (3)	0.053 (3)	0.010 (2)	0.004 (2)	0.011 (2)
C33	0.051 (3)	0.048 (3)	0.047 (3)	0.001 (2)	0.000 (2)	0.013 (2)
C34	0.065 (3)	0.050 (3)	0.040 (3)	0.008 (3)	0.006 (2)	−0.002 (2)
C35	0.053 (3)	0.046 (3)	0.046 (3)	0.010 (2)	0.002 (2)	0.001 (2)
C36	0.085 (4)	0.061 (3)	0.051 (3)	0.013 (3)	−0.010 (3)	0.016 (3)
C37	0.268 (10)	0.137 (7)	0.160 (7)	−0.100 (7)	−0.174 (8)	0.081 (6)
C38	0.226 (9)	0.095 (5)	0.100 (5)	0.050 (6)	−0.066 (5)	0.025 (4)
C39	0.200 (9)	0.369 (15)	0.095 (6)	0.109 (10)	0.062 (6)	0.141 (8)
C40	0.032 (2)	0.042 (3)	0.037 (2)	0.003 (2)	0.0034 (18)	−0.005 (2)
C41	0.051 (3)	0.050 (3)	0.043 (3)	0.005 (2)	−0.012 (2)	−0.005 (2)
C42	0.077 (3)	0.038 (3)	0.055 (3)	0.002 (3)	−0.015 (3)	−0.010 (2)
C43	0.050 (3)	0.044 (3)	0.040 (2)	0.013 (2)	0.003 (2)	0.001 (2)
C44	0.043 (3)	0.050 (3)	0.051 (3)	0.005 (2)	−0.009 (2)	−0.004 (2)
C45	0.037 (2)	0.040 (3)	0.053 (3)	0.005 (2)	−0.003 (2)	−0.003 (2)
C46	0.086 (4)	0.047 (3)	0.060 (3)	0.024 (3)	−0.005 (3)	0.005 (2)
C47	0.227 (9)	0.165 (8)	0.112 (6)	0.157 (7)	0.044 (6)	0.026 (5)
C48	0.164 (7)	0.055 (4)	0.205 (8)	−0.021 (5)	−0.060 (6)	0.053 (5)
C49	0.152 (6)	0.084 (4)	0.085 (4)	0.042 (4)	−0.029 (4)	0.026 (4)
C50	0.034 (2)	0.041 (3)	0.067 (3)	−0.002 (2)	0.003 (2)	0.011 (2)
C51	0.047 (3)	0.067 (4)	0.082 (4)	0.001 (3)	0.005 (3)	0.035 (3)
C52	0.057 (4)	0.088 (5)	0.118 (6)	0.002 (3)	−0.001 (4)	0.062 (5)
C53	0.075 (5)	0.049 (4)	0.184 (9)	0.016 (3)	0.005 (5)	0.035 (5)
C54	0.081 (4)	0.046 (4)	0.149 (7)	0.012 (3)	0.003 (4)	−0.014 (4)
C55	0.056 (3)	0.044 (3)	0.091 (4)	0.005 (3)	0.003 (3)	−0.008 (3)

### 5,11-Bis(4-tert-butylphenyl)-6,12-diphenylnaphthacene (PIFHOC) Geometric parameters (Å, º)

**Table:**

C1—C3	1.348 (6)	C33—C36	1.539 (6)
C1—C2	1.408 (6)	C34—C35	1.392 (6)
C1—H1	0.97 (4)	C34—H34	0.98 (4)
C2—C4	1.355 (6)	C35—H35	0.97 (4)
C2—H2	1.02 (4)	C36—C37	1.464 (7)
C3—C5	1.439 (5)	C36—C39	1.498 (8)
C3—H3	1.02 (4)	C36—C38	1.508 (7)
C4—C6	1.441 (5)	C37—H37A	0.9600
C4—H4	1.01 (4)	C37—H37B	0.9600
C5—C7	1.406 (5)	C37—H37C	0.9600
C5—C6	1.437 (5)	C38—H38A	0.9600
C6—C8	1.402 (5)	C38—H38B	0.9600
C7—C9	1.428 (5)	C38—H38C	0.9600
C7—C20	1.495 (5)	C39—H39A	0.9600
C8—C10	1.431 (5)	C39—H39B	0.9600
C8—C30	1.498 (5)	C39—H39C	0.9600
C9—C11	1.425 (5)	C40—C45	1.384 (5)
C9—C10	1.443 (5)	C40—C41	1.384 (5)
C10—C12	1.422 (5)	C41—C42	1.386 (6)
C11—C13	1.392 (5)	C41—H41	0.98 (4)
C11—C40	1.499 (5)	C42—C43	1.385 (6)
C12—C14	1.402 (5)	C42—H42	0.98 (4)
C12—C50	1.491 (5)	C43—C44	1.373 (6)
C13—C14	1.442 (5)	C43—C46	1.528 (6)
C13—C15	1.442 (5)	C44—C45	1.392 (6)
C14—C16	1.427 (6)	C44—H44	0.95 (4)
C15—C17	1.346 (6)	C45—H45	1.00 (4)
C15—H15	1.01 (4)	C46—C48	1.504 (7)
C16—C18	1.353 (6)	C46—C47	1.519 (7)
C16—H16	0.99 (4)	C46—C49	1.528 (7)
C17—C18	1.412 (7)	C47—H47A	0.9600
C17—H17	1.04 (4)	C47—H47B	0.9600
C18—H18	0.92 (4)	C47—H47C	0.9600
C20—C21	1.385 (6)	C48—H48A	0.9600
C20—C25	1.391 (6)	C48—H48B	0.9600
C21—C22	1.397 (8)	C48—H48C	0.9600
C21—H21	1.00 (5)	C49—H49A	0.9600
C22—C23	1.373 (11)	C49—H49B	0.9600
C22—H22	0.93 (5)	C49—H49C	0.9600
C23—C24	1.344 (11)	C50—C51	1.386 (6)
C23—H23	1.00 (5)	C50—C55	1.401 (6)
C24—C25	1.392 (8)	C51—C52	1.395 (8)
C24—H24	1.00 (5)	C51—H51	1.00 (5)
C25—H25	1.03 (5)	C52—C53	1.374 (9)
C30—C35	1.384 (5)	C52—H52	0.92 (5)
C30—C31	1.388 (5)	C53—C54	1.373 (9)
C31—C32	1.381 (5)	C53—H53	1.02 (5)
C31—H31	0.99 (4)	C54—C55	1.379 (7)
C32—C33	1.387 (6)	C54—H54	1.04 (5)
C32—H32	0.97 (4)	C55—H55	1.06 (5)
C33—C34	1.378 (6)		
			
C3—C1—C2	121.0 (4)	C30—C35—C34	120.5 (4)
C3—C1—H1	121 (3)	C30—C35—H35	121 (3)
C2—C1—H1	118 (3)	C34—C35—H35	118 (3)
C4—C2—C1	120.5 (4)	C37—C36—C39	110.3 (7)
C4—C2—H2	117 (2)	C37—C36—C38	109.7 (6)
C1—C2—H2	123 (2)	C39—C36—C38	104.5 (6)
C1—C3—C5	121.3 (4)	C37—C36—C33	110.7 (4)
C1—C3—H3	120 (2)	C39—C36—C33	109.9 (5)
C5—C3—H3	118 (2)	C38—C36—C33	111.5 (4)
C2—C4—C6	120.8 (4)	C36—C37—H37A	109.5
C2—C4—H4	123 (2)	C36—C37—H37B	109.5
C6—C4—H4	116 (2)	H37A—C37—H37B	109.5
C7—C5—C6	119.9 (4)	C36—C37—H37C	109.5
C7—C5—C3	122.6 (4)	H37A—C37—H37C	109.5
C6—C5—C3	117.5 (4)	H37B—C37—H37C	109.5
C8—C6—C5	119.7 (3)	C36—C38—H38A	109.5
C8—C6—C4	122.0 (4)	C36—C38—H38B	109.5
C5—C6—C4	118.2 (4)	H38A—C38—H38B	109.5
C5—C7—C9	119.5 (3)	C36—C38—H38C	109.5
C5—C7—C20	118.8 (4)	H38A—C38—H38C	109.5
C9—C7—C20	121.2 (3)	H38B—C38—H38C	109.5
C6—C8—C10	119.6 (3)	C36—C39—H39A	109.5
C6—C8—C30	120.4 (3)	C36—C39—H39B	109.5
C10—C8—C30	119.5 (3)	H39A—C39—H39B	109.5
C11—C9—C7	124.0 (3)	C36—C39—H39C	109.5
C11—C9—C10	117.7 (3)	H39A—C39—H39C	109.5
C7—C9—C10	118.4 (3)	H39B—C39—H39C	109.5
C12—C10—C8	122.6 (3)	C45—C40—C41	117.5 (4)
C12—C10—C9	119.2 (3)	C45—C40—C11	122.7 (4)
C8—C10—C9	118.2 (3)	C41—C40—C11	119.8 (3)
C13—C11—C9	120.3 (3)	C40—C41—C42	121.3 (4)
C13—C11—C40	119.3 (3)	C40—C41—H41	118 (3)
C9—C11—C40	119.9 (3)	C42—C41—H41	121 (3)
C14—C12—C10	119.3 (3)	C43—C42—C41	121.5 (4)
C14—C12—C50	119.6 (4)	C43—C42—H42	124 (3)
C10—C12—C50	120.5 (3)	C41—C42—H42	114 (3)
C11—C13—C14	120.1 (3)	C44—C43—C42	116.9 (4)
C11—C13—C15	122.1 (4)	C44—C43—C46	122.1 (4)
C14—C13—C15	117.8 (4)	C42—C43—C46	121.0 (4)
C12—C14—C16	122.5 (4)	C43—C44—C45	122.3 (4)
C12—C14—C13	119.6 (4)	C43—C44—H44	122 (3)
C16—C14—C13	117.9 (4)	C45—C44—H44	116 (3)
C17—C15—C13	121.4 (4)	C40—C45—C44	120.5 (4)
C17—C15—H15	123 (3)	C40—C45—H45	120 (3)
C13—C15—H15	115 (3)	C44—C45—H45	120 (3)
C18—C16—C14	121.2 (4)	C48—C46—C47	109.6 (6)
C18—C16—H16	120 (3)	C48—C46—C49	108.3 (5)
C14—C16—H16	119 (3)	C47—C46—C49	107.0 (5)
C15—C17—C18	120.2 (4)	C48—C46—C43	111.1 (4)
C15—C17—H17	118 (2)	C47—C46—C43	108.6 (4)
C18—C17—H17	122 (2)	C49—C46—C43	112.1 (4)
C16—C18—C17	121.0 (5)	C46—C47—H47A	109.5
C16—C18—H18	123 (3)	C46—C47—H47B	109.5
C17—C18—H18	116 (3)	H47A—C47—H47B	109.5
C21—C20—C25	119.0 (5)	C46—C47—H47C	109.5
C21—C20—C7	122.6 (4)	H47A—C47—H47C	109.5
C25—C20—C7	118.2 (4)	H47B—C47—H47C	109.5
C20—C21—C22	120.2 (6)	C46—C48—H48A	109.5
C20—C21—H21	117 (3)	C46—C48—H48B	109.5
C22—C21—H21	123 (3)	H48A—C48—H48B	109.5
C23—C22—C21	119.4 (7)	C46—C48—H48C	109.5
C23—C22—H22	123 (4)	H48A—C48—H48C	109.5
C21—C22—H22	118 (4)	H48B—C48—H48C	109.5
C24—C23—C22	120.8 (7)	C46—C49—H49A	109.5
C24—C23—H23	118 (3)	C46—C49—H49B	109.5
C22—C23—H23	122 (3)	H49A—C49—H49B	109.5
C23—C24—C25	120.9 (7)	C46—C49—H49C	109.5
C23—C24—H24	126 (3)	H49A—C49—H49C	109.5
C25—C24—H24	113 (3)	H49B—C49—H49C	109.5
C20—C25—C24	119.5 (6)	C51—C50—C55	118.4 (5)
C20—C25—H25	116 (3)	C51—C50—C12	124.2 (4)
C24—C25—H25	124 (3)	C55—C50—C12	117.3 (4)
C35—C30—C31	118.0 (4)	C50—C51—C52	119.8 (6)
C35—C30—C8	118.3 (3)	C50—C51—H51	119 (3)
C31—C30—C8	123.6 (4)	C52—C51—H51	121 (3)
C32—C31—C30	120.7 (4)	C53—C52—C51	120.8 (6)
C32—C31—H31	118 (3)	C53—C52—H52	123 (3)
C30—C31—H31	121 (3)	C51—C52—H52	117 (4)
C31—C32—C33	122.0 (4)	C52—C53—C54	120.0 (6)
C31—C32—H32	117 (3)	C52—C53—H53	119 (3)
C33—C32—H32	121 (3)	C54—C53—H53	121 (3)
C34—C33—C32	116.9 (4)	C53—C54—C55	119.8 (6)
C34—C33—C36	121.2 (4)	C53—C54—H54	121 (3)
C32—C33—C36	121.9 (4)	C55—C54—H54	119 (3)
C33—C34—C35	122.0 (4)	C54—C55—C50	121.2 (6)
C33—C34—H34	121 (3)	C54—C55—H55	120 (3)
C35—C34—H34	117 (3)	C50—C55—H55	118 (3)

## References

[bb1] Bruker (2000). *SMART* and *SAINT*. Bruker AXS Inc., Madison, Wisconsin, USA.

[bb2] Bruker (2016). *APEX3* and *SAINT*. Bruker AXS Inc., Madison, Wisconsin, USA.

[bb3] Clapham, M. L., Murphy, E. C. & Douglas, C. J. (2021). *Synthesis*, **53**, 461–474.

[bb4] Farrugia, L. J. (2012). *J. Appl. Cryst.* **45**, 849–854.

[bb5] Haas, S., Stassen, A. F., Schuck, G., Pernstich, K. P., Gundlach, D. J., Batlogg, B., Berens, U. & Kirner, H. J. (2007). *Phys. Rev. B*, **76**, 115203.

[bb6] Hübschle, C. B., Sheldrick, G. M. & Dittrich, B. (2011). *J. Appl. Cryst.* **44**, 1281–1284. 10.1107/S0021889811043202 PMC3246833 22477785

[bb7] Krause, L., Herbst-Irmer, R., Sheldrick, G. M. & Stalke, D. (2015). *J. Appl. Cryst.* **48**, 3–10. 10.1107/S1600576714022985 PMC4453166 26089746

[bb8] Matsukawa, T., Yoshimura, M., Sasai, K., Uchiyama, M., Yamagishi, M., Tominari, Y., Takahashi, Y., Takeya, J., Kitaoka, Y., Mori, Y. & Sasaki, T. (2010). *J. Cryst. Growth*, **312**, 310–313.

[bb9] Moret, M. & Gavezzotti, A. (2022). *New J. Chem.* **46**, 7626–7637.

[bb10] Ogden, W. A., Ghosh, S., Bruzek, M. J., McGarry, K. A., Balhorn, L., Young, V., Purvis, L. J., Wegwerth, S. E., Zhang, Z., Serratore, N. A., Cramer, C. J., Gagliardi, L. & Douglas, C. J. (2017). *Cryst. Growth Des.* **17**, 643–658.

[bb11] Paraskar, A. S., Reddy, A. R., Patra, A., Wijsboom, Y. H., Gidron, O., Shimon, L. J. W., Leitus, G. & Bendikov, M. (2008). *Chem. Eur. J.* **14**, 10639–10647. 10.1002/chem.200800802 18932176

[bb12] Rigaku (2009). *CrystalClear-SM Expert*. Rigaku Corporation, Tokyo, Japan.

[bb13] Schuck, G., Haas, S., Stassen, A. F., Berens, U. & Batlogg, B. (2007). *Acta Cryst.* E**63**, o2894.

[bb14] Sheldrick, G. M. (2008). *Acta Cryst.* A**64**, 112–122. 10.1107/S0108767307043930 18156677

[bb15] Sheldrick, G. M. (2015*a*). *Acta Cryst.* A**71**, 3–8.

[bb16] Sheldrick, G. M. (2015*b*). *Acta Cryst.* C**71**, 3–8.

[bb17] Sutton, C., Marshall, M. S., Sherrill, C. D., Risko, C. & Brédas, J. L. (2015). *J. Am. Chem. Soc.* **137**, 8775–8782. 10.1021/jacs.5b04066 26075966

[bb18] Uttiya, S., Miozzo, L., Fumagalli, E. M., Bergantin, S., Ruffo, R., Parravicini, M., Papagni, A., Moret, M. & Sassella, A. (2014). *J. Mater. Chem. C.* **2**, 4147–4155.

